# Prevalence and Genetic Diversity of Cross-Assembly Phages in Wastewater Treatment Plants in Riyadh, Saudi Arabia

**DOI:** 10.3390/microorganisms11092167

**Published:** 2023-08-27

**Authors:** Riyadh Alotaibi, Saleh Eifan, Atif Hanif, Islam Nour, Abdulrahman Alkathiri

**Affiliations:** Botany and Microbiology Department, College of Science, King Saud University, Riyadh 11451, Saudi Arabia

**Keywords:** CrAssphage, prevalence, diversity, meteorological impact, Riyadh

## Abstract

The most common DNA virus found in wastewaters globally is the cross-assembly phage (crAssphage). King Saud University wastewater treatment plant (KSU-WWTP); Manfoha wastewater treatment plant (MN-WWTP); and the Embassy wastewater treatment plant (EMB-WWTP) in Riyadh, Saudi Arabia were selected, and 36 untreated sewage water samples during the year 2022 were used in the current study. The meteorological impact on crAssphage prevalence was investigated. CrAssphage prevalence was recorded using PCR and Sanger sequencing. The molecular diversity of crAssphage sequences was studied for viral gene segments from the major capsid protein (MCP) and membrane protein containing the peptidoglycan-binding domain (MP-PBD). KSU-WWTP and EMB-WWTP showed a higher prevalence of crAssphage (83.3%) than MN-WWTP (75%). Phylogenetic analysis of MCP and MP-PBD segments depicted a close relationship to the Japanese isolates. The MCP gene from the current study’s isolate WW/2M/SA/2022 depicted zero evolutionary divergence from 3057_98020, 2683_104905, and 4238_99953 isolates (*d* = 0.000) from Japan. A significant influence of temporal variations on the prevalence of crAssphage was detected in the three WWTPs. CrAssphage displayed the highest prevalence at high temperatures (33–44 °C), low relative humidity (6–14%), and moderate wind speed (16–21 Km/h). The findings provided pioneering insights into crAssphage prevalence and its genetic diversity in WWTPs in Riyadh, Saudi Arabia.

## 1. Introduction

The total number of phages in existence in the world is 10^31^, with over 100 million species, and they are commonly found in contaminated waters such as sewage [1]. It has been found that bacteriophages have the ability and specificity to infect bacteria in various environments [2,3]. The genetic material of over 95% of bacteriophages is ds-DNA, and they are capable of infecting over 130 bacterial genera [4]. In ecosystems of different locations, phages were found in different varieties, depending on their bacterial host diversity [5,6,7]. On the other hand, bacterial diversity can also lead to great diversity of bacteriophages, as reported for *Escherichia coli* [8]. Bacteriophages with enormous genome distributions have the ability to evolve along with their hosts, and studying their genome variations could help us fully understand their evolutionary stages, especially in raw water that has been contaminated [9]. Moreover, the water’s microbial contamination source is primarily fecal and is associated with humans (untreated treatment plants, industrial and livestock wastes, non-collective sewage systems, combined sewage overflow, or wildlife). In untreated wastewater, phages may live free of their bacterial hosts and thus survive longer than bacteria [1]. Thus, phage prevalence and activity are influenced by meteorological and environmental conditions. Several climate factors, including temperature, humidity, wind speed, and rainfall, were reported to affect phage prevalence [10,11]. The cross-assembly phage was discovered from human fecal metagenomics data via computational analysis. It was named after the Cross-Assembly software (http://edwards.sdsu.edu/crass) used to discover crAssphage presence. CrAssphage is a single-stranded, circular DNA virus with a 102 kbp genome. It is present in roughly 50% of individuals from specific human populations and can make up as much as 90% of the total viral DNA load in some individuals’ feces [12]. The members of the bacterial phylum Bacteroidetes make up the majority of the crAssphages’ host range [13]. Since the vast majority of the genes in crAssphage do not correspond to known sequences found in databases, Dutilh highlighted that crAssphage had been ignored in earlier metagenomic research. It was forecasted that a typical crAssphage infects the genus Bacteroides or others present in the human gut needed for the digestion of non-dietary complex polysaccharides [12]. According to an initial study, a few genes were found in the crAssphage genome, which was found with few homologs without any relationship with other phages. A powerful computational assay was utilized to find extended sequence databases for the functions of most of the crAssphage genes. Many crAss-like phages were identified in multiple host-associated and environmental viromes [14]. Genetically, crAssphages are highly diverse and have been grouped into ten genera that share more than 40% of their ORFs [15]. According to the International Committee of Taxonomy of Viruses (ICTV), the Crassvirales order has been classified into four families, ten subfamilies, forty-two genera, and seventy-two species [16,17]. The four subfamilies of the crAss-like phages (Alphacrassvirinae, Betacrassvirinae, Gammacrassvirinae, and Deltacrassvirinae) are distinguished by the proportion of common orthologous genes [18]. The classification was based on phylogenetic analysis of 97 conserved structural genes, including the major capsid protein, the terminase 98 large subunit (terL), and the portal protein [17]. The diversity of crAssphage-positive fecal samples was also shown by using PCR amplification and sequencing of the crAssphage polymerase gene [19]. Stachler et al. created primers to target conserved genomic regions to assess the abundance of crAssphage, while in another study PCR primers were designed for crAssphage detection and diversity evaluation [20,21].

Researchers have looked into the prevalence of crAssphage in a variety of aquatic environments. The continual presence of crAssphage in wastewater has been observed globally with concentrations varying by region. The prevalence of crAssphage in raw wastewater has been reported within a range from 97 to 100 in the USA [22,23,24], 100% in Spain [25], 100% in Australia [26], 100% in the UK [27], and 96% in Italy [28].

The dynamics of viral pathogens are not mimicked by commonly utilized fecal indicator bacteria in aquatic settings or wastewater treatment [29,30,31]. So, direct surveillance of viruses in water systems is vital. CrAssphage was recently found using viral metagenomic study. CrAssphage was detected in sewage but not in animal feces [32]. So, it was concluded that crAssphage could be used as an environmental water pollution microbial source tracking (MST) marker. Studies have also found a high prevalence of crAssphage and a stronger correlation between crAssphage and enteric viruses [27,33,34].

CrAssphage has been found to be highly prevalent in different water sources such as untreated or treated WWTPs, streams, rivers, lakes, and storm drains [35]. As a result, it has been suggested to have great potential for a broad range of applications in fecal virus monitoring as well as MST in environmental waters and wastewater treatment, which may overcome the shortcomings of existing virus control and monitoring methodologies. Our understanding of this phage needs to be enhanced via additional genetic investigation.

Therefore, the current study aimed at molecular detection of crAssphages in raw water of three wastewater treatment plants in the city of Riyadh over the span of one year. Moreover, genetic diversity and seasonal influences on the prevalence of crAssphage were investigated. There is a lack of knowledge regarding crAssphage prevalence and its genetic diversity in Riyadh, Kingdom of Saudi Arabia, and the present study has provided information regarding the circulating crAssphage and its molecular diversity.

## 2. Materials and Methods

### 2.1. Sample Collection

Between January and December 2022, 36 samples of untreated wastewater were collected. Every month, three samples were taken from three various wastewater treatment facilities, including KSU-WWTP (24°43′33.8″ N 46°36′27.9″ E), MN-WWTP (24°35′12.0″ N 46°43′53.0″ E), and EMB-WWTP (24°41′44.1″ N 46°37′34.1″ E) in Riyadh, Saudi Arabia. Samples were collected in sterile 200 mL bottles and transported to the laboratory using a cooler box. On each sampling day, temperature, relative humidity, and wind speed were recorded using information from the Accuweather website (https://accuweather.com).

### 2.2. Viral Concentration

The concentration of crAssphage was performed using the polyethylene glycol (PEG) precipitation method as described previously [36]. Briefly, 200 mL of untreated wastewater sample was added to 25 mL glycine buffer (0.05 M glycine and 0.3 g/L beef extract, PH = 9.6). The mixture was centrifuged at 8000× *g* for 30 min, and the supernatant was filtrated using a 0.22 µm syringe filter. The cleared filtrate was treated with 80 g/L of PEG and 17.5 g/L of NaCl, and the mixture was homogenized at 100 RPM overnight at room temperature. Centrifugation at 13,000× *g* for 30 min was performed, and the supernatant was discarded. The resultant pellets were dissolved with 1 mL of phosphate buffer saline and were stored at −80 °C.

### 2.3. PCR Primer Design

To identify candidate genetic regions for Primer Set Design, selected portions of the prototypical crAssphage ~97 kbp genome (accession number: NC_024711.1) were identified. Sequences from the NCBI database were aligned using MEGA 11 (megasoftware). We focused on predicted coding regions to select sequences of genetic variation. Six PCR primer pairs were designed targeting six structural proteins to amplify selected crAssphage genomic regions. The targeted structural proteins were major capsid protein, membrane protein containing peptidoglycan-binding protein, tail fiber protein, tail-collar fiber protein, tail needle protein, and tail tubular protein. Primer pairs were designed and tested in silico using FAST-PCR 6 (PrimerDigital) and MEGA 11 (Megasoftware). Primer pairs’ specificity and quality were evaluated in vitro using FAST-PCR 6 (PrimerDigital) software, and only two primer sets (MCP and MP-PBD) out of six pairs were selected for in vivo use (Table 1).

### 2.4. DNA Extraction and PCR

CrAssphage DNA was extracted from a 200 µL water sample using the DNeasy PowerWater Kit (Qiagen GmbH, Hilden, Germany), following the manufacturer’s instructions. CrAssphage was detected using 25 µL of 2× Phusion Master Mix (Thermo Fisher Scientific, Waltham, MA, USA) for hexon gene-specific PCR. The 50 μL PCR mixture consisted of a 5 μL DNA template, 1 µL for each, 10 pM forward primer and reverse primer (Table 1), and 18 µL RNase-DNase-free water. The PCR was conducted with an initial denaturation at 94 °C for 60 s, followed by 40 cycles of 94 °C for 30 s, 50 °C for 30 s, and 72 °C for 30 s, and a final extension at 72 °C for 10 min.

### 2.5. Agarose Gel Electrophoresis

PCR products were examined in 1.5% agarose gel. In a glass flask, 0.75 g of agarose were dissolved in 50 mL of 1X Tris-Acetate-EDTA (TAE) buffer solution. The mixture was heated for one minute in the microwave. The gel casting tray was assembled, and the comb was placed above the tray’s surface. Ethidium bromide 4 μL (10 mg/mL) was added to the agarose mixture after it had cooled to ≈40 °C. Afterward, the gel was poured into the casting tray and allowed to solidify at room temperature. The electrophoresis chamber was filled with 1× TAE buffer, and the gel was placed in the electrophoresis chamber. Sample wells were loaded with 10 μL of PCR products mixed with 2 μL (abm Canada, Richmond, BC, Canada, cat#G030) of loading dye. Six µL of DNA ladder (Gilpilot^®^ Wide Range Ladder 100 bps cat#239125) was loaded, and the apparatus lid was connected to the power source. The electrophoresis was performed at 100 volts for 30 min. The gel was visualized and photographed using the gel documentation system (BIO-RAD, Hercules, CA, USA).

### 2.6. Amplicon Purification and Sequencing

PCR product electrophoresis was performed on 2% Agarose gel, and 371 bp and 223 bp amplicons were purified using the Wizard^®^ SV Gel and PCR Clean-Up System (Promega Co., Madison, WI, USA), according to the instructions of the manufacturer. The cleaned amplicons were sequenced using the BigDye Terminator v3.1 cycle sequencing kit (Applied Biosystems, Foster City, CA, USA) in both forward and reverse directions (4X). Sequences were generated using the ABI genetic analyzer 3130Xl (Applied Biosystems^®^, Carlsbad, CA, USA).

### 2.7. Phylogenetic Analysis

The sequences were cleaned using Bioedit 7.2 (Nucleics Co., Sydney, Australia) and analyzed with MEGA X [37]. The sequence multiple alignments were performed using ClustalW keeping the default settings (15 opening penalty and 6.66 extension penalty). The phylogenetic trees were constructed and aligned with the best-fitting nucleotide substitution model relying on the minimum Bayesian information criterion. The reliability of the phylogenetic tree was estimated via the bootstrapping of 1000 replicates. The genetic distances were calculated using the Kimura three-parameter method.

### 2.8. Statistical Analysis

Pearson’s correlation coefficient matrix was utilized for the determination of probable relationships among the sampling areas. One-way analysis of variance was performed to assess the influence and significance of high- and low-temperature ranges on crAssphage prevalence. Linear curve-fitting was used to estimate the relationships between different sampling areas (as dependent variables) and high and low temperatures, humidity, and wind speed (as independent variables). All statistical analyses were performed using the XL-STAT statistical package software (Ver. 2019, Excel Add-ins soft SARL, New York, NY, USA). A *p*-value of < 0.05 was considered significant.

### 2.9. Prevalence of crAssphage in Different Sampling Areas

The prevalence of crAssphage was calculated using the following equation:

$$\mathrm{Prevalence}\;=\;\frac{\mathrm{Number}\;\mathrm{of}\;\mathrm{positive}\;\mathrm{samples}}{\mathrm{Number}\;\mathrm{of}\;\mathrm{total}\;\mathrm{samples}}\times 100$$

## 3. Results

### 3.1. Prevalence of crAssphage in Different Sampling Areas

**Figure 1:**
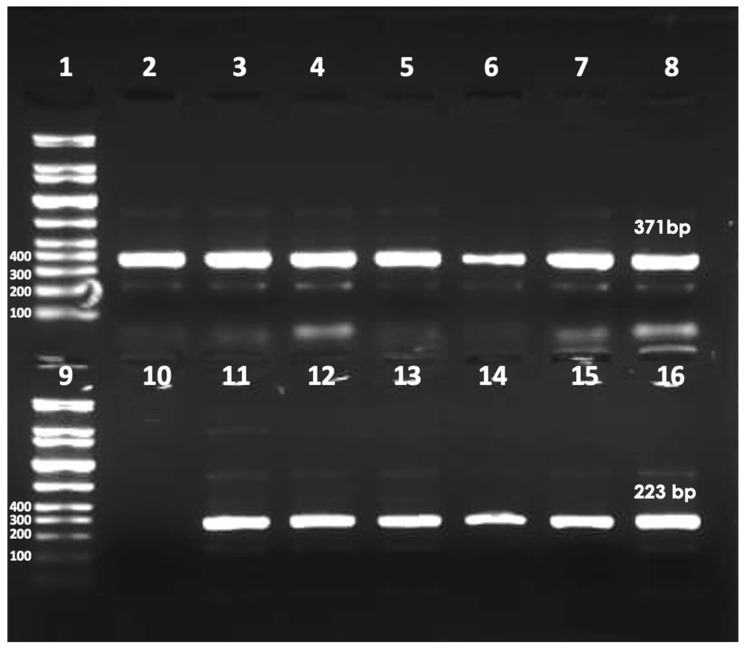
PCR products gel image. Lanes 1 and 9, DNA Ladder GelPilot 100 bp Plus Ladder. Lanes 2 to 8371 bp crAssphage MCP amplicons. Lane 10, negative control (nuclease-free water). Lanes 11 to 16,223 bp crAssphage MP-PBD amplicons.

The 371 bp and 223 bp amplicons were detected in 29 (80.5%) of the samples (Figure 1). KSU-WWTP and EMB-WWTP had the highest prevalence of crAssphage (83.3%), whereas MN-WWTP had the lowest prevalence (75%) (Table 2).

### 3.2. Phylogenetic Analysis for the MCP Gene Sequences

**Figure 2:**
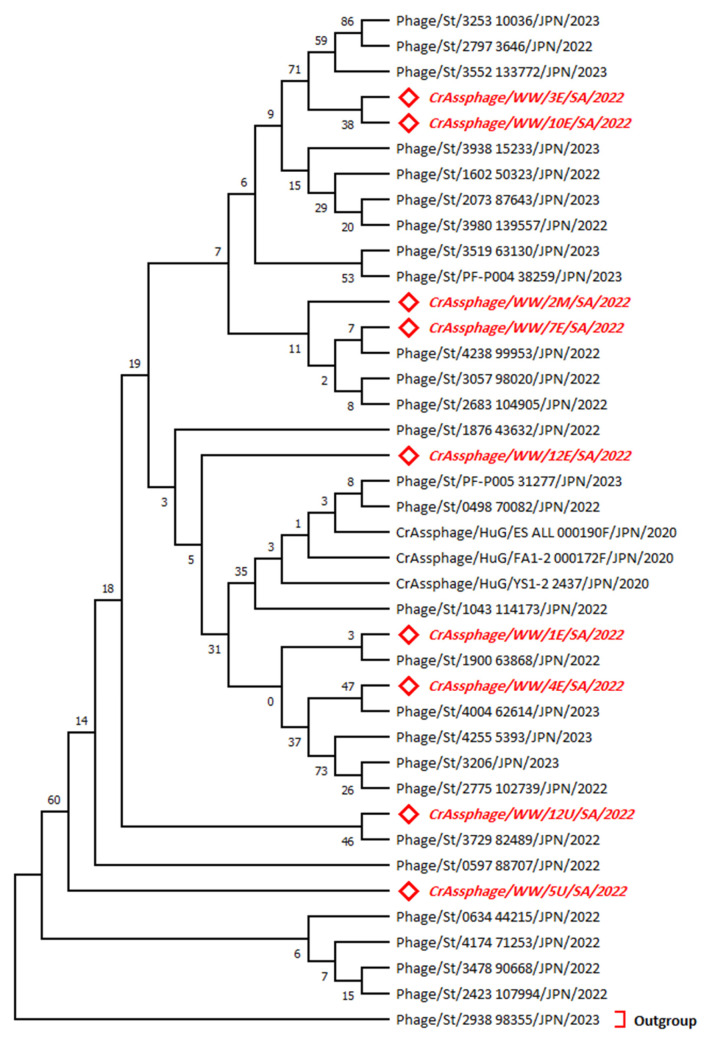
Phylogenetic tree for the MCP gene sequences constructed using the maximum likelihood method and the Tamura 3-parameter model. The red italicized sequences indicate the current study sequences. Outgroup refers to the highest divergent sequence. The taxa are abbreviated to the formulation: Species/Source/Isolate/Country/Year.

A phylogenetic tree (Figure 2) was constructed based on the nucleotide sequences of the major capsid gene region in all nine field isolates of crAssphage. The highest log likelihood tree is displayed (−702.50). The percentage of trees in which the associated taxa clustered together are provided at each branch. The rate variation model allowed for some sites to be evolutionarily invariable ([+*I*], 47.71% sites), according to the best-fitting substitution model validation. The horizontal distance is expressed as the number of nucleotide substitutions per site. The isolate 2938_98355 was used as an outgroup. Accession numbers of sequences used for the phylogenetic analysis are displayed in the Supplementary Materials.

The genetic tree analysis for the MCP gene showed the isolate clustering to other isolates from Japan. Interestingly, some isolates of the MCP gene show zero distance (*d* = 0.00) in evolutionary divergence with isolates from Japan 3057_98020, 2683_104905, and 4238_99953, as shown in the Supplementary Data.

### 3.3. Phylogenetic Analysis for the MP-PBD Gene Sequences

**Figure 3:**
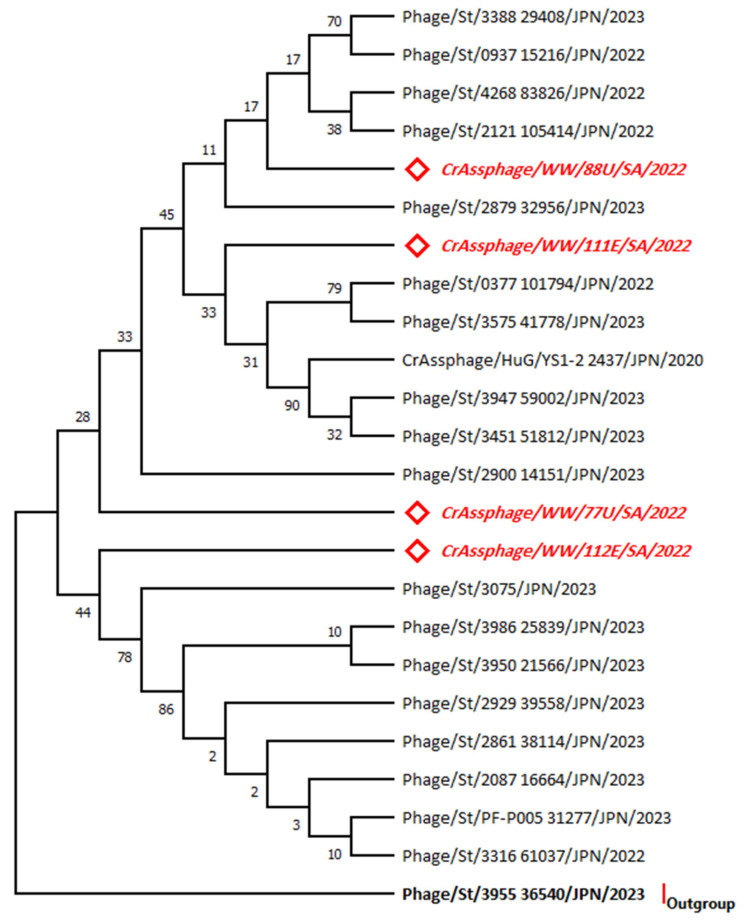
Phylogenetic tree for the MP-PBD gene sequences constructed using the maximum likelihood method and the Tamura 3-parameter model. The red italicized sequences indicate the current study sequences. Outgroup refers to the highest divergent sequence. The taxa abbreviated to the formulation: Species/Source/Isolate/Country/Year.

A phylogenetic tree (Figure 3) was constructed based on the nucleotide sequences of the membrane protein containing a peptidoglycan-binding protein region in all four isolates of crAssphage. The highest log likelihood tree is displayed (−457.77). The percentage of trees in which the associated taxa clustered together are provided at each branch. The rate variation model allowed for some sites to be evolutionarily invariable ([+*I*], 60.25% sites), according to the best-fitting substitution model validation. There were a total of 223 positions in the final dataset. The horizontal distance is expressed as the number of nucleotide substitutions per site. The isolate 3955_36450 was used as an outgroup. Accession numbers of sequences used for the phylogenetic analysis are displayed in the Supplementary Materials. All samples positive for crAssphage were sequenced and compared to sequences available in the GenBank database with the BLAST tool. The genetic tree analysis for the MP-PBD gene showed the isolate clustering to other isolates from Japan. The crAssphage 88U isolate showed a distance of (*d* = 0.0100) in evolutionary divergence with the 3388 29408, 0937 15216, and 2121 105414 isolates (Supplementary Data).

### 3.4. Meteorological Influences on crAssphage Prevalence

The prevalence of phages varied according to the location of WWTPs and the meteorological conditions. The prevalence of crAssphage was significantly affected by the high-temperature factor (*p* = <0.0001) and humidity factor (*p* = 0.0004). However, low temperature and wind speed showed no significant effect.

### 3.5. Temperature Variation’s Role on crAssphage Prevalence

**Figure 4:**
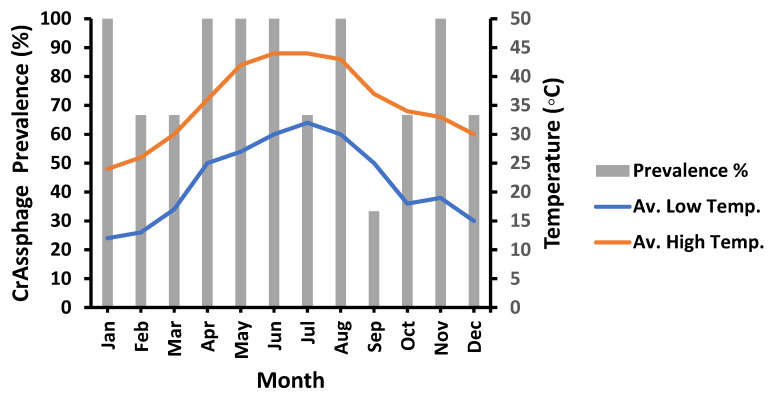
Effect of temperature variation on crAssphage prevalence. Av. High Temp., the average high temperature of the sampling day. Av. Low Temp., the average low temperature of the sampling day.

The impact of temperature on the prevalence of crAssphage was investigated by linking the prevalence and the average high or low temperature of the sampling day. According to the data presented in Figure 4, the prevalence of crAssphage was found to be 100% in January, April, May, June, August, and November at the three sampling locations. In contrast, the lowest crAssphage prevalence (33%) was detected in September with temperature ranges of (25–37 °C).

### 3.6. Humidity Variations Impact on crAssphage Prevalence

**Figure 5:**
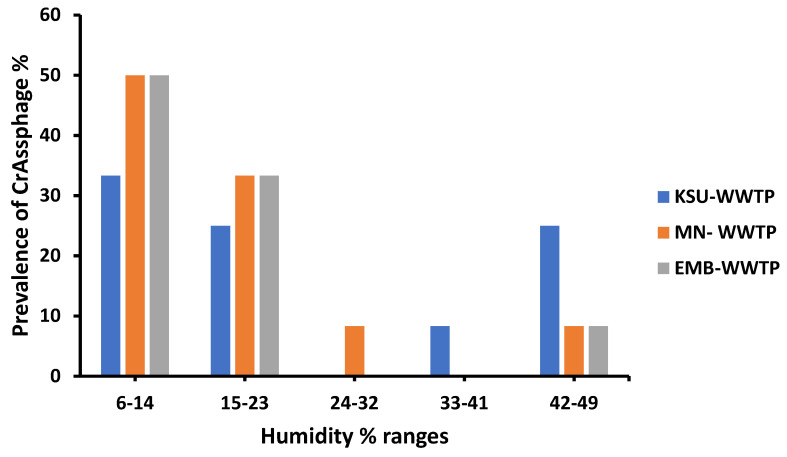
Prevalence of crAssphage in different humidity ranges.

In all sampling locations, crAssphage favored the lowest relative humidity ranges (6–14%), with a prevalence of 50% in MN and EMB WWTPs and a prevalence of 33.3% in KSU WWTP (Figure 5). On the other hand, crAssphage was not detected at the humidity range (24–32) in both KSU-WWTP and EMB-WWTP. Also, crAssphage was not detected in MN-WWTP and EMB-WWTP in the (33–41) humidity range (Figure 5). At different humidity ranges, crAssphage prevalence significantly varied, highlighting the importance of relative humidity in determining crAssphage prevalence in raw water from WWTPs (*p* < 0.0004; Table 3).

### 3.7. Wind Speed Influence on the Prevalence of crAssphage

**Figure 6:**
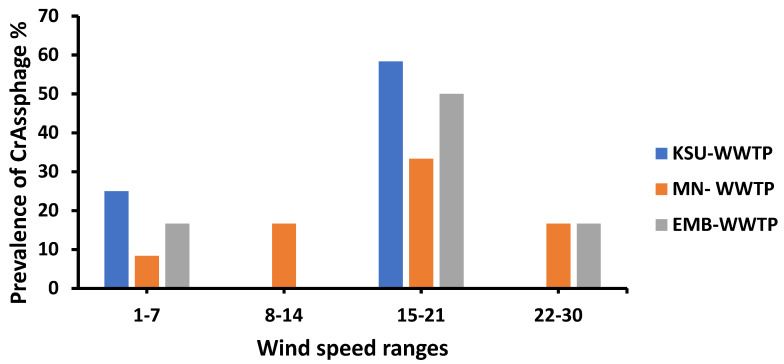
CrAssphage prevalence at different wind speed ranges.

The prevalence of crAssphage varied with wind speed ranges. The highest crAssphage prevalence was detected at a relatively high wind speed range (15–21 km/h) in all three sampling areas. On the other hand, crAssphage was not detected in KSU-WWTP and EMB-WWTP in the (8–14) wind speed ranges (Figure 6). CrAssphage prevalence was detected at different wind speed levels varying from a low wind speed of 1–7 Km/h to a high wind speed level of 30 Km/h. Therefore, wind speed did not significantly influence crAssphage prevalence (R^2^ = 0.03; Table 3).

## 4. Discussion

CrAssphage has been discovered recently, and the widespread nature of this phage indicates that it is quite old but still not well studied [38]. The crAssphage is a highly abundant bacteriophage found in wastewater [24,28,39,40,41]; however, its molecular diversity in waste water is not well documented. In the current study, we investigated the prevalence of crAssphage and its molecular diversity in Riyadh with seasonal influences.

Based on the detection of both MCP 371 bp and MP-PBD 223 bp amplicons, crAssphage prevalence was found (80.5%). Out of 36 untreated wastewater samples, a total of 29 were found positive via PCR. KSU-WWTP and EMB-WWTP showed (83.3%) prevalence of crAssphage, while MN-WWTP depicted a prevalence of 75%. A study in Florida investigated the prevalence of crAssphage in untreated wastewater. CrAssphage prevalence was 100% out of eight samples collected [22]. Another study explored the prevalence of crAssphage in New Orleans, USA. Out of 13 samples from untreated wastewater, crAssphage was detected in all the samples tested, 13/13 (100%) [42]. The samples were taken monthly for a 13-month period between March 2017 and March 2018. In addition, a study completed in the United Kingdom found the prevalence of crAssphage to be 100% over a one-year period. A total of 49 out of 49 samples were found positive for crAssphage in untreated wastewater [27]. Untreated wastewater samples were collected from August 2016 to August 2017 at the four major wastewater treatment plants. A study in Japan investigated the prevalence of crAssphage in different water sources such as rivers, raw sewage, secondary treated sewage, and effluent. CrAssphage prevalence was 100% in untreated wastewater, 12/12 (100%). Samples were collected between August and December 2016 from Yamanashi Prefecture, Japan [34].

Untreated wastewater samples from a US WWTP were evaluated for their fecal source tracking marker removal efficiency and found to be 100% [41]. Similar to this, all 12 raw wastewater samples tested in Australia were found positive for crAssphage with an average of 6.43 ± 0.14 log10GC/100 mL [26]. In Spain, 23 samples of wastewater were tested for the presence of crAssphage using primers targeting distinct regions of the crAssphage genome. All samples were reported positive at a range of 5.4 × 10^6.9^ log10GC/100 mL [25]. In Thailand, 21 sewage samples were tested, and all of them tested positive for crAssphage with a concentration range from 5.28 to 7.38 log10GC/100 mL [39]. The prevalence of crAssphage in Italian wastewater was studied for several years, 2014 (n = 43), 2015 (n = 34), 2016 (n = 49), 2017 (n = 26), and 2018 (n = 4). Samples of untreated wastewater were obtained from 25 WWTPs located throughout Italy. CrAssphage was detected in 150 out of 156 samples (96%) [28]. This study is in line with our results; we detected crAssphage in 29 out of 36 samples in Riyadh wastewater as compared to the other reports. There could be a number of causes for this. First of all, this study’s scope differs from previous comparable investigations that measured viral concentrations in wastewater using direct PCR. A natural variation in the crAssphage prevalence that was not depicted in previous data may have been captured in the current study via the increased scope, which included one-year sampling attempts and targeting different genetic segments [43,44]. Moreover, different studies employ various concentration techniques, which could affect the observed prevalence of crAssphage. The negative samples in the current study may contain lower concentrations of crAssphage below the limits of detection. Lastly, new research has demonstrated the great diversity of crAss-like phages that can affect the detection of target genes using PCR [45,46].

It is likely that the PCR used here did not detect the natural diversity in crAss-like phages, since it targeted the MCP and MP-PBD regions of crAssphage. Eventually, additional data would be helpful to conclude the findings of the current study, with a cautious assumption that wastewater is always contaminated with crAssphage.

In previous studies, most authors studied the polymerase gene for genotyping of crAssphages, since they are relatively considered conserved genes, whereas, in the current study, we designed our own primer using available sequences in NCBI. Basically, we choose a highly variable region of two genes (MCP and the MP-PBD) by aligning the sequences downloaded from NCBI for the selection of primers. Both genes showed a close relationship to other isolates from Japan. Other studies reported the diversity of major gene encoding DNA polymerase [12,19]. Based on their sequence identities, their isolates were found close to China’s isolates. The study showed that the crAssphage strains described have different genome characteristics compared to the strains in the United States. A study in Korea investigated DNA sequencing and phylogenetic analysis of the crAssphage, which targeted the major gene encoding DNA polymerase; all domestic crAssphage sequences showed a close relationship to the USA isolate p-crAssphage (NC_024711), 71.9% (23/32) for genotype II, and 28.1% (9/32) for genotype I. In addition, the distribution of these domestic crAssphage genotypes was found to be similar to that in China. CrAssphage diversity also depends on four potential host genera (Bacteroides, Prevotella, Porphyromonas, and Parabacteroides) [47]. Similarly, in a wastewater environment, crAssphage diversity and prevalence depend on the host range and population. This could be a reason why we found various types of crAssphages in different types of WWTPs. Specifically, we could not obtain results during some months, such as February, March, July, September, October, and December, in some WWTP locations. This could be explained in various ways; likewise, the impact of rains, human activities, and industrial waste influence the WWTPs of the Riyadh region, which can affect bacterial host and crAssphage populations.

In the current study, the high-temperature variations were found to significantly influence the prevalence of crAssphage, whereas another study investigated the effect of temperature on crAssphage abundance and suggested that high temperature had a strong negative correlation with crAssphage concentration [33].

In the current study, there was no significant influence of humidity on the prevalence of crAssphage in the three different sampling areas. Another study investigated the prevalence of crAssphage, and humidity was considered as a parameter in their study. During the collection days, daily relative humidity ranged from 36% to 86%. CrAssphage depicted no significant impact on prevalence [40].

Furthermore, crAssphage abundance correlates more strongly with enteric viruses than other fecal markers [27,33,34,41,48]. CrAssphage has the potential to replace the shortcomings in existing virus monitoring and control methodologies in fecal virus monitoring and microbial source tracking in wastewater. CrAssphage prevalence usually displayed non-significant seasonal patterns. So, it suggests that the use of crAssphage as a fecal contamination indicator enables the assessment of pollution.

## 5. Conclusions

This study highlighted the prevalence and the molecular diversity of crAssphage in wastewaters of Riyadh. Further research is needed to exploit the crAssphage genomes to identify geographic differences and diversity. Additionally, crAssphage has excellent potential as a virus performance indicator for determining and ensuring virus reduction in wastewater treatment processes.

## Figures and Tables

**Table 1:** List of primers for specific PCR detection of crAssphage.

Target Genes	Primers	Amplicon Size	Reference
MCP	F: 5′-TGACCGTGATACTCAAGATG-3′ R: 5′-GACGTACTATACGAACATTCTG-3′	371 bp	Current study
MP-PBD	F: 5′-CCTGTTACTRATTCTACTRC-3′ R: 5′-ATTCWTRAAGAGTTCTACGAATCC-3′	223 bp	

**Table 2:** CrAssphage prevalence in sewage water of different WWTPs.

Sampling Location	CrAssphage +ve	CrAssphage Prevalence %
KSU-WWTP	10	83.3%
MN-WWTP	9	75%
EMB-WWTP	10	83.3%

**Table 3:** Statical analysis of environmental factors on prevalence of crAssphage.

Environmental Factor	R^2^	RMSE	Equation
High temperature (T_H_)	0.9526 *	1.8982	%PrevCrAss = −10.1247 + 0.992 × T_H_
Low temperature (T_L_)	0.1629	6.94868	%PrevCrAss = 12.211 + 0.417 × T_L_
Relative humidity (RH%)	0.62089 **	9.8919	%PrevCrAss = 38.333 − 0.9259 × RH%
Wind speed (WS)	0.03	17.404	%PrevCrAss = 16.0317 + 0.357 × WS

** significant at *p* < 0.0004, * significant at *p* < 0.0001. %PrevCrAss refers to the crAssphage percentage at different sampling areas. RSME denotes the root mean square error, which is an absolute measure of fit.

## Data Availability

The sequences used in this study for phylogenetic analysis are openly available in the NCBI Gen-Bank repository with accession numbers mentioned.

## Supplementary Materials

The following supporting information can be downloaded at: https://www.mdpi.com/article/10.3390/microorganisms11092167/s1.
