# Supplementary material for: Prevalence and Genetic Diversity of Cross-Assembly Phages in Wastewater Treatment Plants in Riyadh, Saudi Arabia
Source: Microorganisms. 2023 Aug 27;11(9):2167. doi: 10.3390/microorganisms11092167 (PMC10535421; doi:10.3390/microorganisms11092167)
Supplement: Supplementary file 1 [file microorganisms-11-02167-s001.zip › Primer selection for MCP and MP-PBD.pdf]

MCP

Forward primer

TGACCGTGATACTCAAGATG

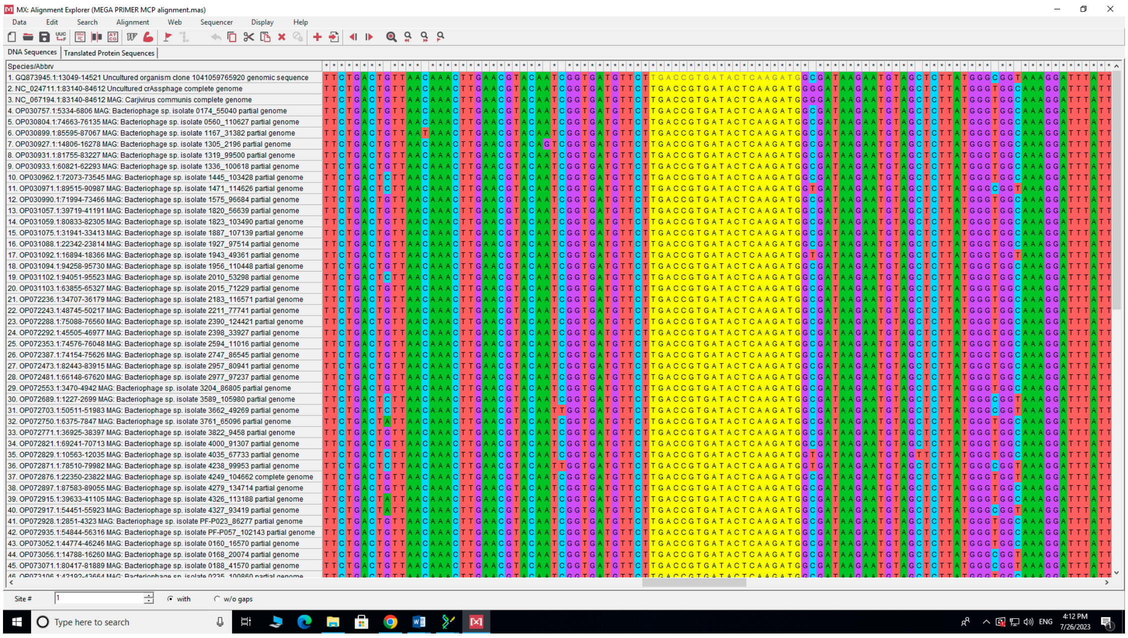

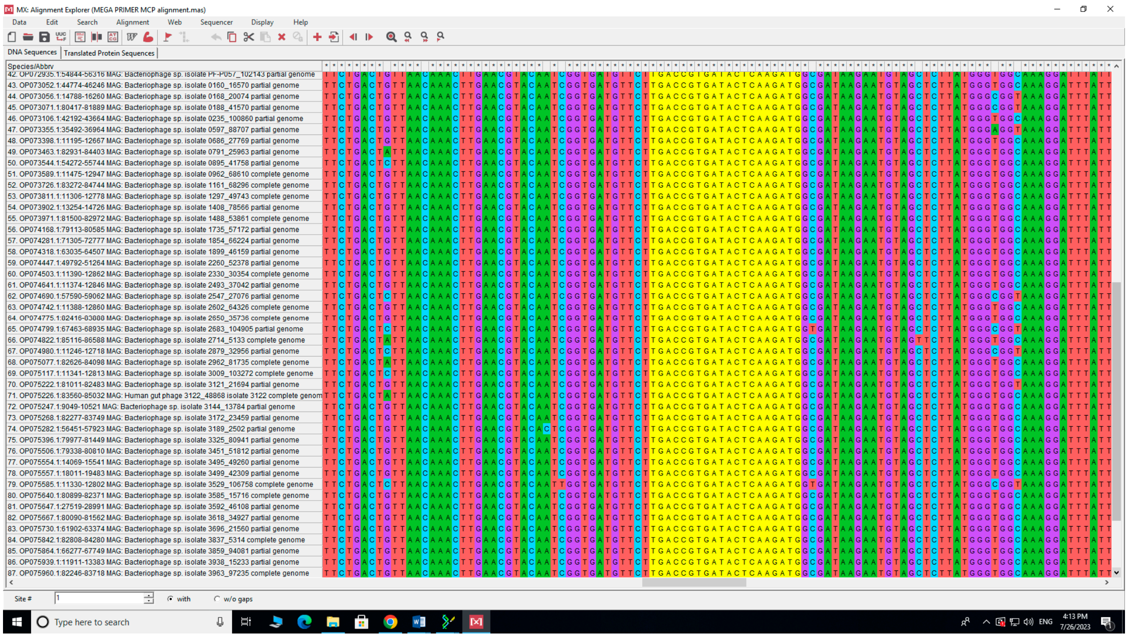

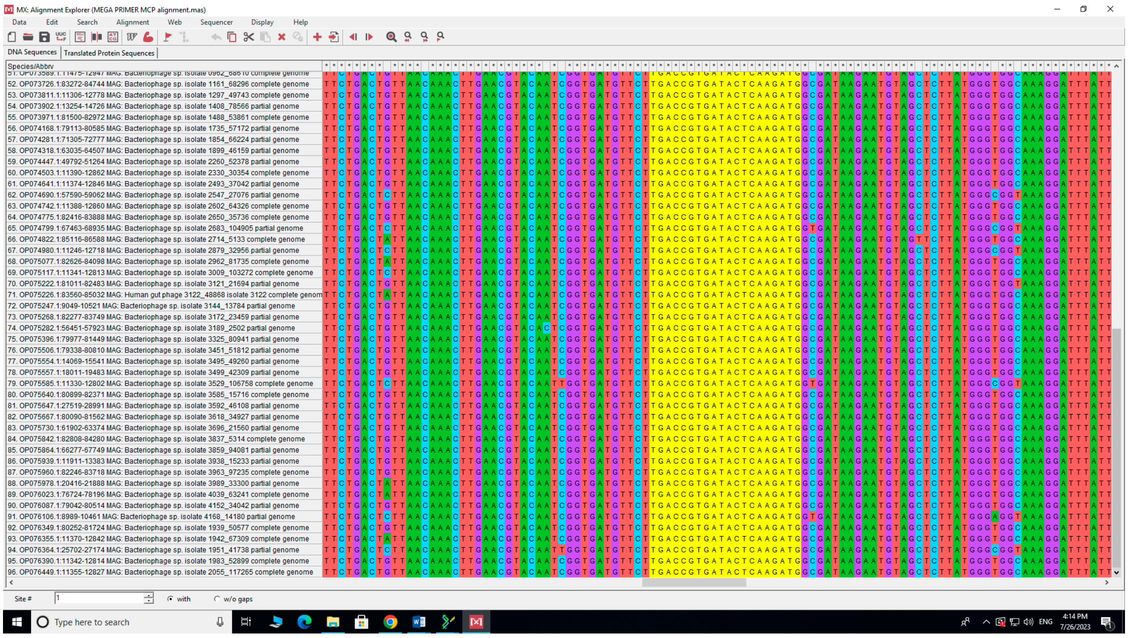
MX: Alignment Explorer (MEGA PRIMER MCP alignment.mas)
Data
Edit
Search
Alignment
Web
Sequencer
Display
Help
DNA Sequences
Translated Protein Sequences
Species/Abbrv

MCP

Reverse primer

GACGTACTATACGAACATTCTG

(Reverse complementary)

CAGAATGTTCGTATAGTACGTC

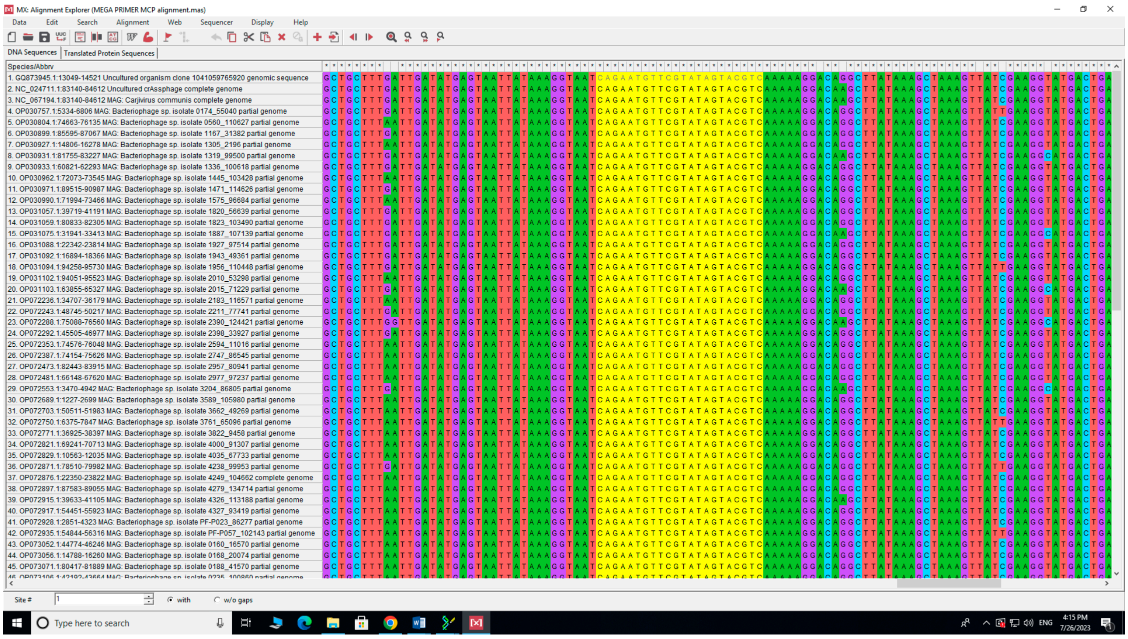

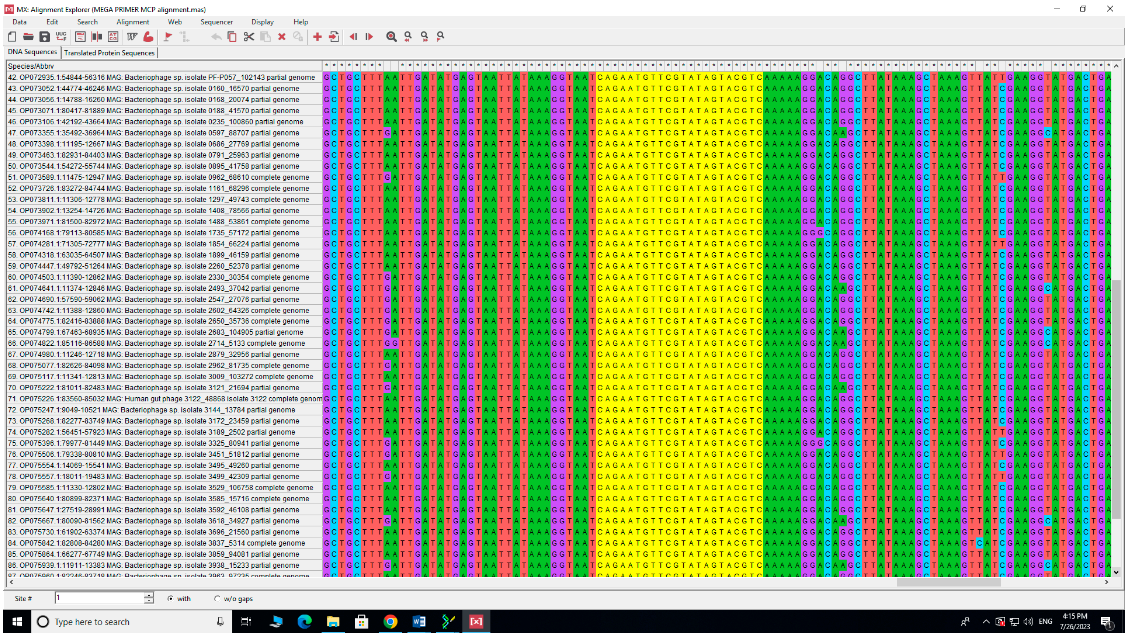

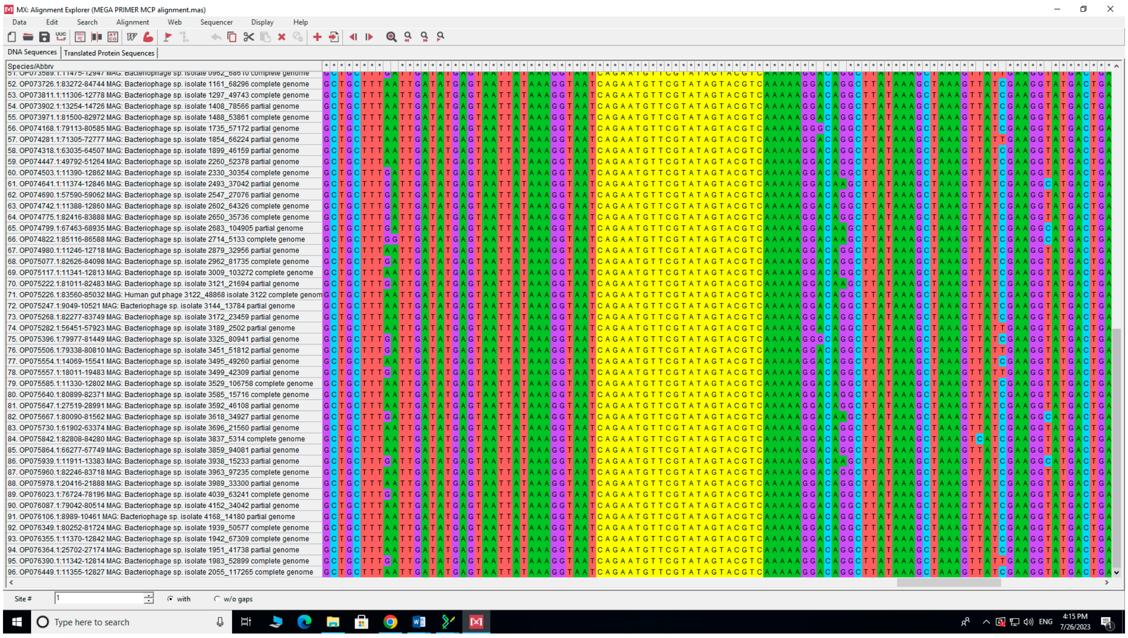
MX: Alignment Explorer (MEGA PRIMER MCP alignment.mas)
Data Edit Search Alignment Web Sequencer Display Help
DNA Sequences
Translated Protein Sequences
Species/Abbrv
52. OP073726.1:83272-84744 MAG: Bacteriophage sp. isolate 1161_68296 complete genome
53. OP073811.1:11306-12778 MAG: Bacteriophage sp. isolate 1297_49743 complete genome
54. OP073902.1:13254-14726 MAG: Bacteriophage sp. isolate 1408_78566 partial genome
55. OP073971.1:81500-82972 MAG: Bacteriophage sp. isolate 1488_53861 complete genome
56. OP074168.1:79113-80585 MAG: Bacteriophage sp. isolate 1735_57172 partial genome
57. OP074281.1:71305-72777 MAG: Bacteriophage sp. isolate 1854_66224 partial genome
58. OP074318.1:63035-64507 MAG: Bacteriophage sp. isolate 1899_46159 partial genome
59. OP074447.1:49792-51264 MAG: Bacteriophage sp. isolate 2260_52378 partial genome
60. OP074503.1:11390-12862 MAG: Bacteriophage sp. isolate 2330_30354 complete genome
61. OP074641.1:11374-12846 MAG: Bacteriophage sp. isolate 2493_37042 partial genome
62. OP074690.1:57590-59062 MAG: Bacteriophage sp. isolate 2547_27076 partial genome
63. OP074742.1:11388-12860 MAG: Bacteriophage sp. isolate 2602_64326 complete genome
64. OP074775.1:82416-83888 MAG: Bacteriophage sp. isolate 2650_35736 complete genome
65. OP074799.1:67463-68935 MAG: Bacteriophage sp. isolate 2683_104905 partial genome
66. OP074822.1:85116-86588 MAG: Bacteriophage sp. isolate 2714_5133 complete genome
67. OP074980.1:11246-12718 MAG: Bacteriophage sp. isolate 2879_32956 partial genome
68. OP075077.1:82626-84098 MAG: Bacteriophage sp. isolate 2962_81735 complete genome
69. OP075117.1:11341-12813 MAG: Bacteriophage sp. isolate 3009_103272 complete genome
70. OP075222.1:81011-82483 MAG: Bacteriophage sp. isolate 3121_21694 partial genome
71. OP075226.1:83560-85032 MAG: Human gut phage 3122_48868 isolate 3122 complete genom
72. OP075247.1:9049-10521 MAG: Bacteriophage sp. isolate 3144_13784 partial genome
73. OP075268.1:82277-83749 MAG: Bacteriophage sp. isolate 3172_23459 partial genome
74. OP075282.1:56451-57923 MAG: Bacteriophage sp. isolate 3189_2502 partial genome
75. OP075396.1:79977-81449 MAG: Bacteriophage sp. isolate 3325_80941 partial genome
76. OP075506.1:79338-80810 MAG: Bacteriophage sp. isolate 3451_51812 partial genome
77. OP075554.1:14069-15541 MAG: Bacteriophage sp. isolate 3495_49260 partial genome
78. OP075557.1:18011-19483 MAG: Bacteriophage sp. isolate 3499_42309 partial genome
79. OP075585.1:11330-12802 MAG: Bacteriophage sp. isolate 3529_106758 complete genome
80. OP075640.1:80899-82371 MAG: Bacteriophage sp. isolate 3585_15716 complete genome
81. OP075647.1:27519-28991 MAG: Bacteriophage sp. isolate 3592_46108 partial genome
82. OP075667.1:80090-81562 MAG: Bacteriophage sp. isolate 3618_34927 partial genome
83. OP075730.1:61902-63374 MAG: Bacteriophage sp. isolate 3696_21560 partial genome
84. OP075842.1:82808-84280 MAG: Bacteriophage sp. isolate 3837_5314 complete genome
85. OP075864.1:66277-67749 MAG: Bacteriophage sp. isolate 3859_94081 partial genome
86. OP075939.1:11911-13383 MAG: Bacteriophage sp. isolate 3938_15233 partial genome
87. OP075960.1:82246-83718 MAG: Bacteriophage sp. isolate 3963_97235 complete genome
88. OP075978.1:20416-21888 MAG: Bacteriophage sp. isolate 3989_33300 partial genome
89. OP076023.1:76724-78196 MAG: Bacteriophage sp. isolate 4039_63241 complete genome
90. OP076087.1:79042-80514 MAG: Bacteriophage sp. isolate 4152_34042 partial genome
91. OP076106.1:8989-10461 MAG: Bacteriophage sp. isolate 4168_14180 partial genome
92. OP076349.1:80252-81724 MAG: Bacteriophage sp. isolate 1939_50577 complete genome
93. OP076355.1:11370-12842 MAG: Bacteriophage sp. isolate 1942_67309 complete genome
94. OP076364.1:25702-27174 MAG: Bacteriophage sp. isolate 1951_41738 partial genome
95. OP076390.1:11342-12814 MAG: Bacteriophage sp. isolate 1983_52899 complete genome
96. OP076449.1:11355-12827 MAG: Bacteriophage sp. isolate 2055_117265 complete genome
Site #
with
w/o gaps
Type here to search

MP-PBD

Forward primer

CCTGTTACYGATTCTACTGC

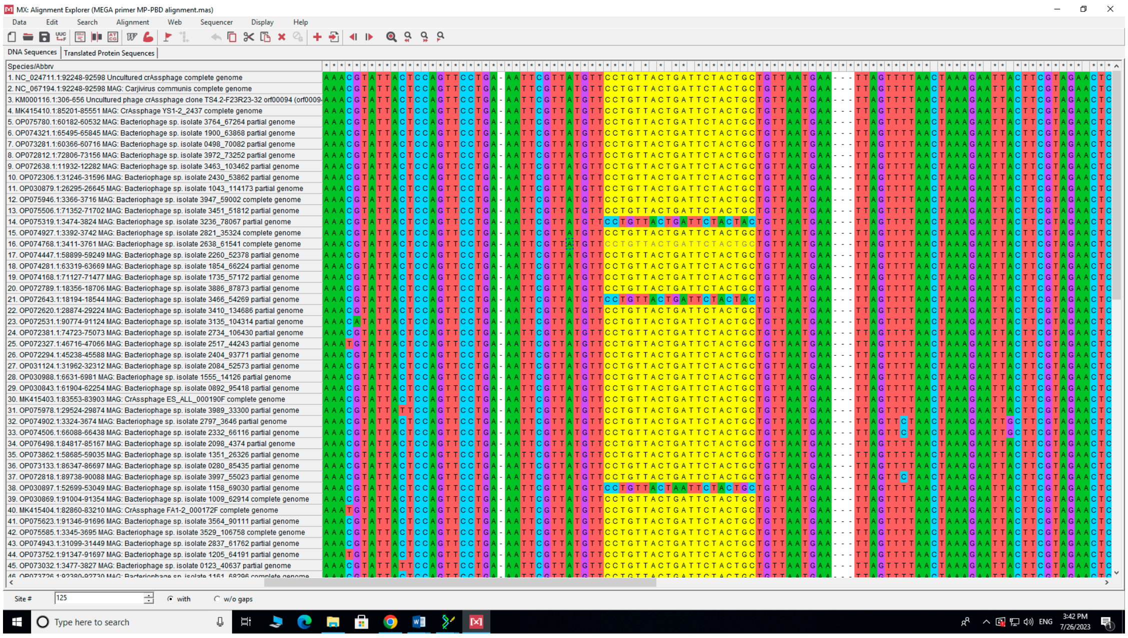

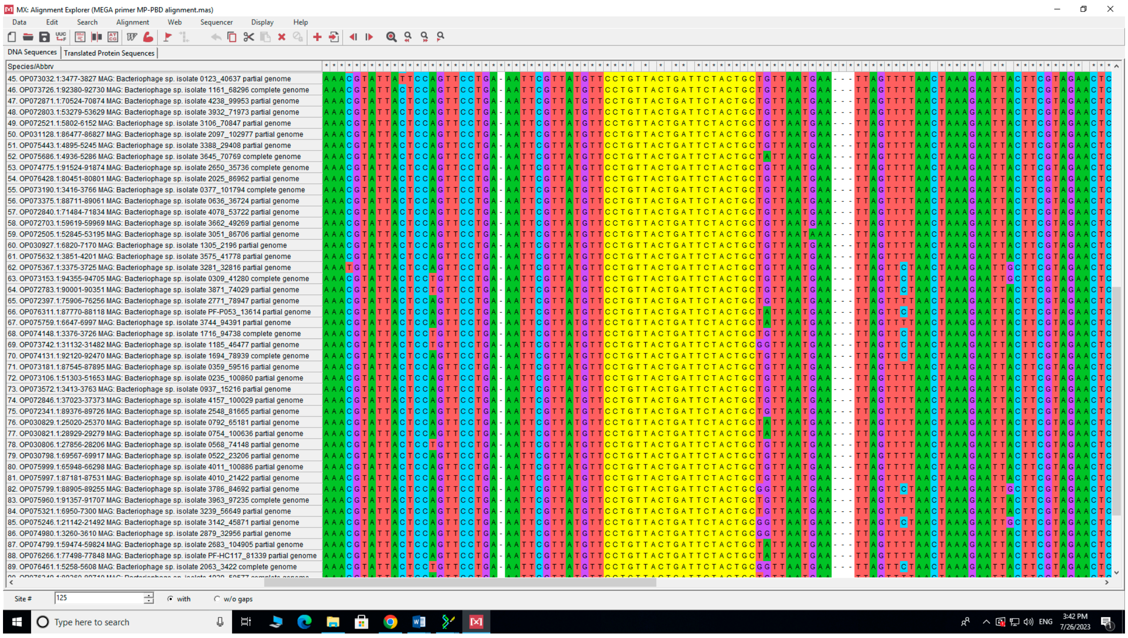

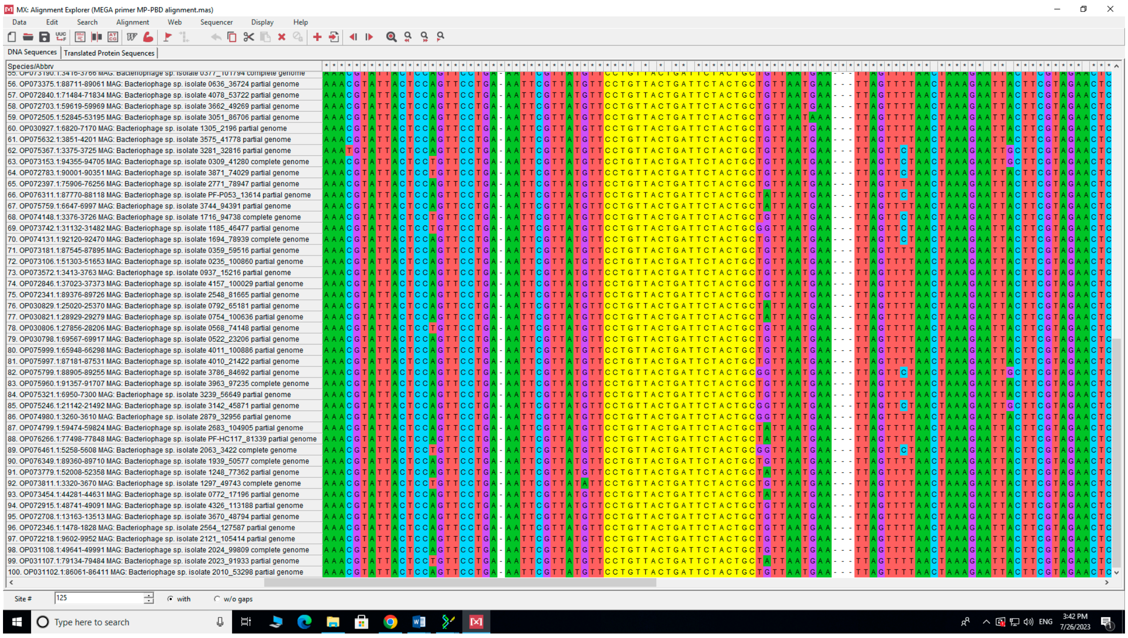

MP-PBD

Reverse primer

ATTCWTRAAGAGTTCTACGAATCC

(Reverse complementary)

GGATTCGTAGAACTCTTRAWGAAT

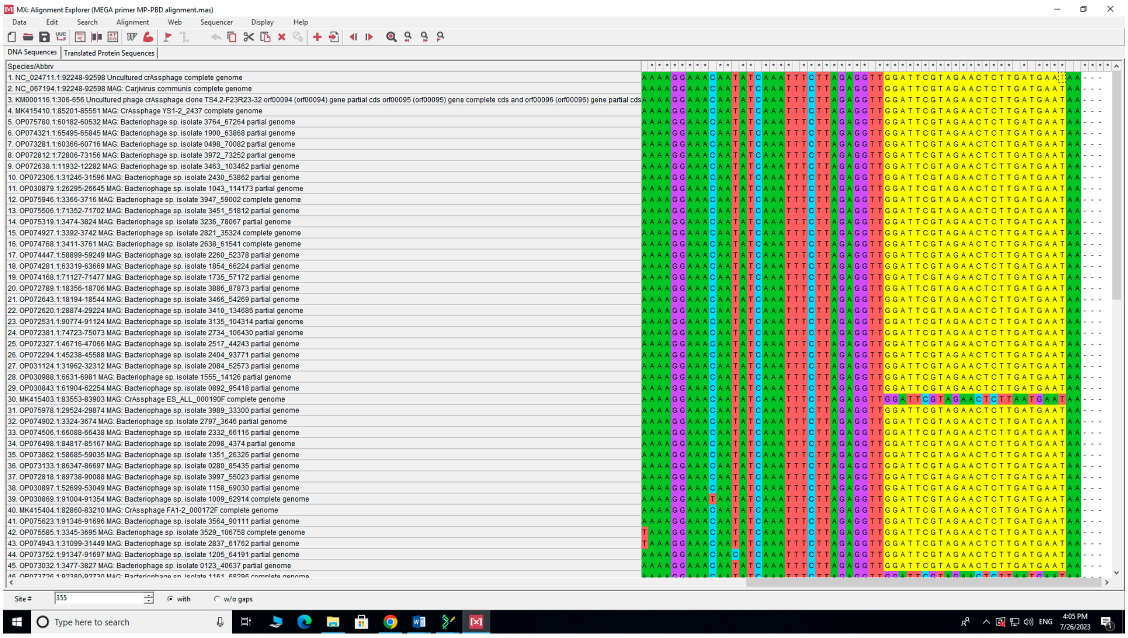
M✕: Alignment Explorer (MEGA primer MP-PBD alignment.mas)
Data Edit Search Alignment Web Sequencer Display Help
DNA Sequences
Translated Protein Sequences
Species/Abbrv
Site #
Type here to search

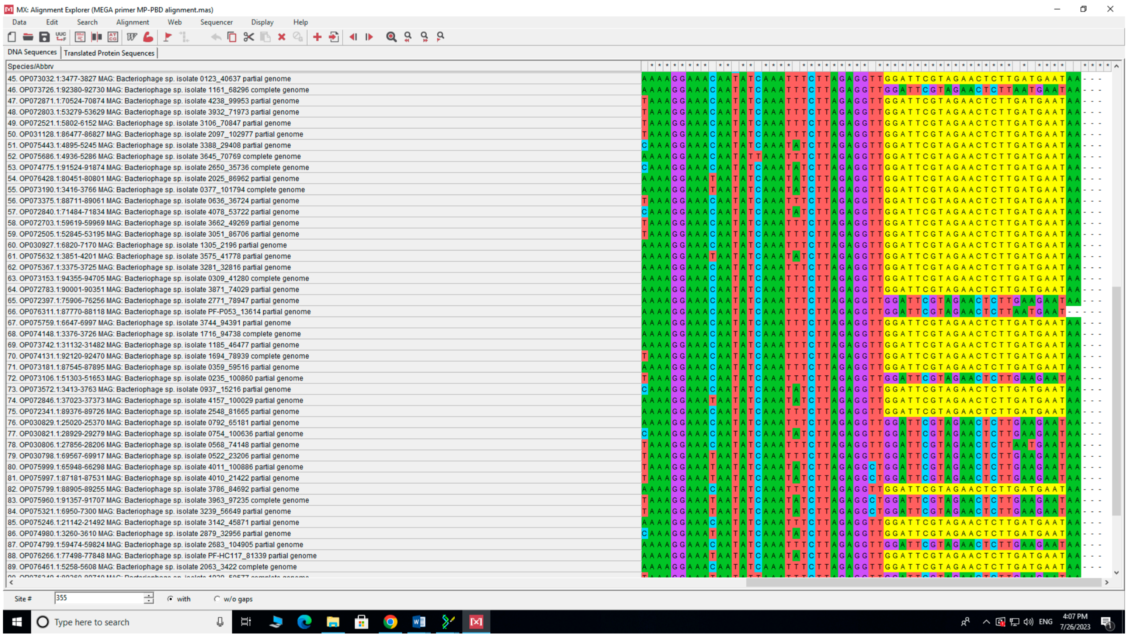
M✕: Alignment Explorer (MEGA primer MP-PBD alignment.mas)
Data Edit Search Alignment Web Sequencer Display Help
DNA Sequences
Translated Protein Sequences
Species/Abbrv
Site #
Type here to search

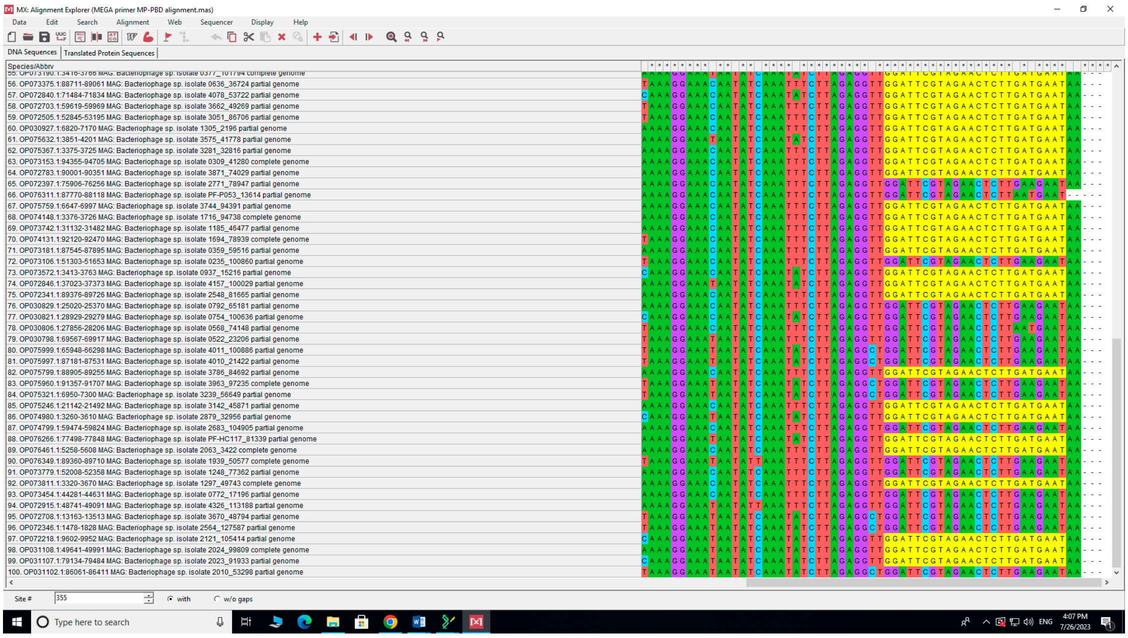
MX: Alignment Explorer (MEGA primer MP-PBD alignment.mas)
Data
Edit
Search
Alignment
Web
Sequencer
Display
Help
DNA Sequences
Translated Protein Sequences
Species/Abbrv
56. OP073375.1:88711-89061 MAG: Bacteriophage sp. isolate 0636_36724 partial genome
57. OP072840.1:71484-71834 MAG: Bacteriophage sp. isolate 4078_53722 partial genome
58. OP072703.1:59619-59969 MAG: Bacteriophage sp. isolate 3662_49269 partial genome
59. OP072505.1:52845-53195 MAG: Bacteriophage sp. isolate 3051_86706 partial genome
60. OP030927.1:6820-7170 MAG: Bacteriophage sp. isolate 1305_2196 partial genome
61. OP075632.1:3851-4201 MAG: Bacteriophage sp. isolate 3575_41778 partial genome
62. OP075367.1:3375-3725 MAG: Bacteriophage sp. isolate 3281_32816 partial genome
63. OP073153.1:94355-94705 MAG: Bacteriophage sp. isolate 0309_41280 complete genome
64. OP072783.1:90001-90351 MAG: Bacteriophage sp. isolate 3871_74029 partial genome
65. OP072397.1:75906-76256 MAG: Bacteriophage sp. isolate 2771_78947 partial genome
66. OP076311.1:87770-88118 MAG: Bacteriophage sp. isolate PF-P053_13614 partial genome
67. OP075759.1:6647-6997 MAG: Bacteriophage sp. isolate 3744_94391 partial genome
68. OP074148.1:3376-3726 MAG: Bacteriophage sp. isolate 1716_94738 complete genome
69. OP073742.1:31132-31482 MAG: Bacteriophage sp. isolate 1185_46477 partial genome
70. OP074131.1:92120-92470 MAG: Bacteriophage sp. isolate 1694_78939 complete genome
71. OP073181.1:87545-87895 MAG: Bacteriophage sp. isolate 0359_59516 partial genome
72. OP073106.1:51303-51653 MAG: Bacteriophage sp. isolate 0235_100860 partial genome
73. OP073572.1:3413-3763 MAG: Bacteriophage sp. isolate 0937_15216 partial genome
74. OP072846.1:37023-37373 MAG: Bacteriophage sp. isolate 4157_100029 partial genome
75. OP072341.1:89376-89726 MAG: Bacteriophage sp. isolate 2548_81665 partial genome
76. OP030829.1:25020-25370 MAG: Bacteriophage sp. isolate 0792_65181 partial genome
77. OP030821.1:28929-29279 MAG: Bacteriophage sp. isolate 0754_100636 partial genome
78. OP030806.1:27856-28206 MAG: Bacteriophage sp. isolate 0568_74148 partial genome
79. OP030798.1:69567-69917 MAG: Bacteriophage sp. isolate 0522_23206 partial genome
80. OP075999.1:65948-66298 MAG: Bacteriophage sp. isolate 4011_100886 partial genome
81. OP075997.1:87181-87531 MAG: Bacteriophage sp. isolate 4010_21422 partial genome
82. OP075799.1:88905-89255 MAG: Bacteriophage sp. isolate 3786_84692 partial genome
83. OP075960.1:91357-91707 MAG: Bacteriophage sp. isolate 3963_97235 complete genome
84. OP075321.1:6950-7300 MAG: Bacteriophage sp. isolate 3239_56649 partial genome
85. OP075246.1:21142-21492 MAG: Bacteriophage sp. isolate 3142_45871 partial genome
86. OP074980.1:3260-3610 MAG: Bacteriophage sp. isolate 2879_32956 partial genome
87. OP074799.1:59474-59824 MAG: Bacteriophage sp. isolate 2683_104905 partial genome
88. OP076266.1:77498-77848 MAG: Bacteriophage sp. isolate PF-HC117_81339 partial genome
89. OP076461.1:5258-5608 MAG: Bacteriophage sp. isolate 2063_3422 complete genome
90. OP076349.1:89360-89710 MAG: Bacteriophage sp. isolate 1939_50577 complete genome
91. OP073779.1:52008-52358 MAG: Bacteriophage sp. isolate 1248_77362 partial genome
92. OP073811.1:3320-3670 MAG: Bacteriophage sp. isolate 1297_49743 complete genome
93. OP073454.1:44281-44631 MAG: Bacteriophage sp. isolate 0772_17196 partial genome
94. OP072915.1:48741-49091 MAG: Bacteriophage sp. isolate 4326_113188 partial genome
95. OP072708.1:13163-13513 MAG: Bacteriophage sp. isolate 3670_48794 partial genome
96. OP072346.1:1478-1828 MAG: Bacteriophage sp. isolate 2564_127587 partial genome
97. OP072218.1:9602-9952 MAG: Bacteriophage sp. isolate 2121_105414 partial genome
98. OP031108.1:49641-49991 MAG: Bacteriophage sp. isolate 2024_99809 complete genome
99. OP031107.1:79134-79484 MAG: Bacteriophage sp. isolate 2023_91933 partial genome
100. OP031102.1:86061-86411 MAG: Bacteriophage sp. isolate 2010_53298 partial genome
Site #
355
with
w/o gaps
