# Supplementary material for: Prevalence and Genetic Diversity of Cross-Assembly Phages in Wastewater Treatment Plants in Riyadh, Saudi Arabia
Source: Microorganisms. 2023 Aug 27;11(9):2167. doi: 10.3390/microorganisms11092167 (PMC10535421; doi:10.3390/microorganisms11092167)
Supplement: Supplementary file 1 [file microorganisms-11-02167-s001.zip › supplementary 1.pdf]

Table S1. Best fitting nucleotide substitution model for MCP gene

Model	#Param	BIC	AICc	lnL	Invariant	Gamma	R	Freq A	Freq T	Freq C	Freq G
T92+G+I	81	2182.718804	1567.607324	-702.3536008	0.47708895	0.05	2.40287187	0.324359838	0.324359838	0.175640162	0.175640162
T92+G	80	2194.96806	1587.439646	-713.2807691	n/a	0.05	2.35310058	0.324359838	0.324359838	0.175640162	0.175640162
HKY+G+I	83	2199.080457	1568.803663	-700.9293455	0.47708895	0.05	2.43588235	0.309433962	0.339285714	0.143665768	0.207614555
TN93+G+I	84	2200.665357	1562.806317	-696.919255	0.47708895	0.05	2.69371397	0.309433962	0.339285714	0.143665768	0.207614555
HKY+G	82	2209.639805	1586.94553	-711.0115603	n/a	0.05	2.39655355	0.309433962	0.339285714	0.143665768	0.207614555
TN93+G	83	2209.833026	1579.556231	-706.30563	n/a	0.05	2.74692616	0.309433962	0.339285714	0.143665768	0.207614555
K2+G+I	80	2214.74469	1607.216277	-723.1690842	0.47708895	0.05	2.37541268	0.25	0.25	0.25	0.25
T92+I	80	2219.051271	1611.522858	-725.3223749	0.47708895	n/a	2.3213131	0.324359838	0.324359838	0.175640162	0.175640162
JC+G+I	79	2219.388231	1619.443159	-730.2933955	0.47708895	0.05	0.5	0.25	0.25	0.25	0.25
GTR+G+I	87	2221.697112	1561.092981	-693.0275099	0.47708895	0.05	2.53460908	0.309433962	0.339285714	0.143665768	0.207614555
K2+G	79	2222.483714	1622.538642	-731.8411369	n/a	0.05	2.31776354	0.25	0.25	0.25	0.25
JC+G	78	2230.621863	1638.260407	-740.7127522	n/a	0.05	0.5	0.25	0.25	0.25	0.25
T92	79	2235.59135	1635.646279	-738.394955	n/a	n/a	2.28154836	0.324359838	0.324359838	0.175640162	0.175640162
GTR+G	86	2239.45725	1586.434542	-706.7101197	n/a	0.05	1.57291656	0.309433962	0.339285714	0.143665768	0.207614555
HKY+I	82	2240.465695	1617.77142	-726.4245053	0.47708895	n/a	2.32751727	0.309433962	0.339285714	0.143665768	0.207614555
TN93+I	83	2242.051554	1611.77476	-722.4148941	0.47708895	n/a	2.38725577	0.309433962	0.339285714	0.143665768	0.207614555
K2+I	79	2248.145444	1648.200373	-744.6720023	0.47708895	n/a	2.28447512	0.25	0.25	0.25	0.25
HKY	81	2250.229329	1635.117848	-736.1088629	n/a	n/a	2.30631884	0.309433962	0.339285714	0.143665768	0.207614555
TN93	82	2252.27421	1629.579935	-732.3287626	n/a	n/a	2.30861883	0.309433962	0.339285714	0.143665768	0.207614555
JC+I	78	2256.007261	1663.645806	-753.4054515	0.47708895	n/a	0.5	0.25	0.25	0.25	0.25
K2	78	2263.912646	1671.55119	-757.3581438	n/a	n/a	2.27898953	0.25	0.25	0.25	0.25
GTR+I	86	2271.275119	1618.25241	-722.6190542	0.47708895	n/a	1.52578608	0.309433962	0.339285714	0.143665768	0.207614555
JC	77	2271.866501	1687.088935	-766.1376122	n/a	n/a	0.5	0.25	0.25	0.25	0.25
GTR	85	2281.328756	1635.887745	-732.4484137	n/a	n/a	1.52495106	0.309433962	0.339285714	0.143665768	0.207614555

Models with the lowest BIC scores (Bayesian Information Criterion) are considered to describe the substitution pattern the best. For each model, AICc value (Akaike Information Criterion, corrected), the Maximum Likelihood value (lnL), and the number of parameters (including branch lengths) are also presented. Non-uniformity of evolutionary rates among sites may be modeled by using a discrete Gamma distribution (+G) with 5 rate categories and by assuming that a certain fraction of sites is evolutionarily invariable (+I). Whenever applicable, estimates of gamma shape parameters and/or the estimated fraction of invariant sites are shown.

*Abbreviations: TR: General Time Reversible; HKY: Hasegawa-Kishino-Yano; TN93: Tamura-Nei; T92: Tamura 3-parameter; K2: Kimura 2-parameter; JC: Jukes-Cantor

Table S2. Sequences used for phylogenetic analysis of CrAssphage MCP gene.

Accession number	Sequence nomination	Abbreviated name used for phylogenetic tree	Country	Source
**MK415410.1**	MAG: CrAssphage YS1-2_2437, complete genome	CrAssphage/HuG/YS1-2_2437/JPN/2020	Japan	human gut
**MK415404.1**	MAG: CrAssphage FA1-2_000172F, complete genome	CrAssphage/HuG/FA1-2_000172F/JPN/2020	Japan	human gut
**MK415403.1**	MAG: CrAssphage ES_ALL_000190F, complete genome	CrAssphage/HuG/ES_ALL_000190F/JPN/2020	Japan	human gut
**OP075997.1**	MAG: Bacteriophage sp. isolate 4010_21422, partial genome	Phage/St/4010_21422/JPN/2023	Japan	Stool
**OP074799.1**	MAG: Bacteriophage sp. isolate 2683_104905, partial genome	Phage/St/2683_104905/JPN/2022	Japan	Stool
**OP074720.1**	MAG: Bacteriophage sp. isolate 2574_5239, complete genome	Phage/St/2574_5239/JPN/2022	Japan	Stool
**OP074321.1**	MAG: Bacteriophage sp. isolate 1900_63868, partial genome	Phage/St/1900_63868/JPN/2022	Japan	Stool
**OP076277.1**	MAG: Bacteriophage sp. isolate PF-P005_31277, partial genome	Phage/St/PF-P005_31277/JPN/2023	Japan	Stool
**OP073281.1**	MAG: Bacteriophage sp. isolate 0498_70082, partial genome	Phage/St/0498_70082/JPN/2022	Japan	Stool
**OP072871.1**	MAG: Bacteriophage sp. isolate 4238_99953, partial genome	Phage/St/4238_99953/JPN/2022	Japan	Stool
**OP072508.1**	MAG: Bacteriophage sp. isolate 3057_98020, partial genome	Phage/St/3057_98020/JPN/2022	Japan	Stool
**OP074943.1**	MAG: Bacteriophage sp. isolate 2837_61762, partial genome	Phage/St/2837_61762/JPN/2023	Japan	Stool
**OP075029.1**	MAG: Bacteriophage sp. isolate 2924_82214, complete genome	Phage/St/2924_82214/JPN/2023	Japan	Stool

**OP074295.1**	MAG: Bacteriophage sp. isolate 1876_43632, partial genome	Phage/St/1876_43632/JPN/2022	Japan	Stool
**OP076471.1**	MAG: Bacteriophage sp. isolate 2073_87643, complete genome	Phage/St/2073_87643/JPN/2023	Japan	Stool
**OP076275.1**	MAG: Bacteriophage sp. isolate PF-P004_38259, complete genome	Phage/St/PF-P004_38259/JPN/2023	Japan	Stool
**OP073779.1**	MAG: Bacteriophage sp. isolate 1248_77362, partial genome	Phage/St/1248_77362/JPN/2022	Japan	Stool
**OP074030.1**	MAG: Bacteriophage sp. isolate 1563_44488, complete genome	Phage/St/1563_44488/JPN/2022	Japan	Stool
**OP072814.1**	MAG: Bacteriophage sp. isolate 3980_139557, partial genome	Phage/St/3980_139557/JPN/2022	Japan	Stool
**OP072735.1**	MAG: Bacteriophage sp. isolate 3729_82489, partial genome	Phage/St/3729_82489/JPN/2022	Japan	Stool
**OP072612.1**	MAG: Bacteriophage sp. isolate 3394_67887, partial genome	Phage/St/3394_67887/JPN/2022	Japan	Stool
**OP075973.1**	MAG: Bacteriophage sp. isolate 3979_5654, complete genome	Phage/St/3979_5654/JPN/2023	Japan	Stool
**OP075609.1**	MAG: Bacteriophage sp. isolate 3552_133772, partial genome	Phage/St/3552_133772/JPN/2023	Japan	Stool
**OP075575.1**	MAG: Bacteriophage sp. isolate 3519_63130, complete genome	Phage/St/3519_63130/JPN/2023	Japan	Stool
**OP076364.1**	MAG: Bacteriophage sp. isolate 1951_41738, partial genome	Phage/St/1951_41738/JPN/2023	Japan	Stool
**OP076307.1**	MAG: Bacteriophage sp. isolate PF-P050_93161, complete genome	Phage/St/PF-P050_93161/JPN/2023	Japan	Stool
**OP074053.1**	MAG: Bacteriophage sp. isolate 1602_50323, partial genome	Phage/St/1602_50323/JPN/2022	Japan	Stool
**OP075587.1**	MAG: Bacteriophage sp. isolate 3531_60876, partial genome	Phage/St/3531_60876/JPN/2023	Japan	Stool
**MW067002.1**	CrAssphage sp. C0526BW15, complete genome	CrAssphage/St/C0526BW15/RSA/2020	South Africa	Stool
**OP076106.1**	MAG: Bacteriophage sp. isolate 4168_14180, partial genome	Phage/St/4168_14180/JPN/2023	Japan	Stool

**OP075991.1**	MAG: Bacteriophage sp. isolate 4004_62614, complete genome	Phage/St/4004_62614/JPN/2023	Japan	Stool
**OP075298.1**	MAG: Human gut phage 3206_30171 isolate 3206, complete genome	Phage/St/3206/JPN/2023	Japan	Stool
**OP075072.1**	MAG: Bacteriophage sp. isolate 2959_55556, complete genome	Phage/St/2959_55556/JPN/2023	Japan	Stool
**OP074885.1**	MAG: Bacteriophage sp. isolate 2775_102739, partial genome	Phage/St/2775_102739/JPN/2022	Japan	Stool
**MT006214.1**	CrAssphage LMMB, complete genome	CrAssphage/HuG/LMMB/RUS/2020	Russia	human gut
**OP075053.1**	MAG: Bacteriophage sp. isolate 2938_98355, complete genome	Phage/St/2938_98355/JPN/2023	Japan	Stool
OP073373.1	MAG: Bacteriophage sp. isolate 0634_44215, partial genome	Phage/St/0634_44215/JPN/2022	Japan	Stool
**OP073355.1**	MAG: Bacteriophage sp. isolate 0597_88707, partial genome	Phage/St/0597_88707/JPN/2022	Japan	Stool
**OP072853.1**	MAG: Bacteriophage sp. isolate 4174_71253, partial genome	Phage/St/4174_71253/JPN/2022	Japan	Stool
**OP072840.1**	MAG: Bacteriophage sp. isolate 4078_53722, partial genome	Phage/St/4078_53722/JPN/2022	Japan	Stool
**OP072650.1**	MAG: Bacteriophage sp. isolate 3478_90668, partial genome	Phage/St/3478_90668/JPN/2022	Japan	Stool
**OP072305.1**	MAG: Bacteriophage sp. isolate 2423_107994, partial genome	Phage/St/2423_107994/JPN/2022	Japan	Stool
**OP031092.1**	MAG: Bacteriophage sp. isolate 1943_49361, partial genome	Phage/St/1943_49361/JPN/2022	Japan	Stool
**NC_067194.1**	MAG: Carjivirus communis, complete genome	CrAssphage/St/Carjivirus communis/USA/2023	USA	Stool
**OP075939.1**	MAG: Bacteriophage sp. isolate 3938_15233, partial genome	Phage/St/3938_15233/JPN/2023	Japan	Stool
**OP076023.1**	MAG: Bacteriophage sp. isolate 3618_34927, partial genome	Phage/St/4039_63241/JPN/2023	Japan	Stool
**OP075667.1**	MAG: Bacteriophage sp. isolate 3618_34927, partial genome	Phage/St/3618_34927/JPN/2023	Japan	Stool

**MK238400.1**	CrAssphage ZA, complete genome	CrAssphage/St/ZA/RSA/2019	South Africa	Stool
**OP074690.1**	MAG: Bacteriophage sp. isolate 2547_27076, partial genome	Phage/St/2547_27076/JPN/2022	Japan	Stool
**OP030754.1**	MAG: Bacteriophage sp. isolate 0138_31247, partial genome	Phage/St/0138_31247/JPN/2022	Japan	Stool
**OP075981.1**	MAG: Bacteriophage sp. isolate 3993_101156, complete genome	Phage/St/3993_101156/JPN/2023	Japan	Stool
**OP075724.1**	MAG: Bacteriophage sp. isolate 3689_37730, partial genome	Phage/St/3689_37730/JPN/2023	Japan	Stool
**OP075432.1**	MAG: Bacteriophage sp. isolate 3376_53066, partial genome	Phage/St/3376_53066/JPN/2023	Japan	Stool
**OP075342.1**	MAG: Bacteriophage sp. isolate 3253_10036, complete genome	Phage/St/3253_10036/JPN/2023	Japan	Stool
**OP075276.1**	MAG: Bacteriophage sp. isolate 3184_74577, partial genome	Phage/St/3184_74577/JPN/2023	Japan	Stool
**OP074902.1**	MAG: Bacteriophage sp. isolate 2797_3646, partial genome	Phage/St/2797_3646/JPN/2022	Japan	Stool
**OP072294.1**	MAG: Bacteriophage sp. isolate 2404_93771, partial genome	Phage/St/2404_93771/JPN/2022	Japan	Stool
**OP030879.1**	MAG: Bacteriophage sp. isolate 1043_114173, partial genome	Phage/St/1043_114173/JPN/2022	Japan	Stool
**OP030856.1**	MAG: Bacteriophage sp. isolate 0963_33341, partial genome	Phage/St/0963_33341/JPN/2022	Japan	Stool
**OP076186.1**	MAG: Bacteriophage sp. isolate 4255_5393, partial genome	Phage/St/4255_5393/JPN/2023	Japan	Stool
**OP074053.1**	MAG: Bacteriophage sp. isolate 1602_50323, partial genome	Phage/St/1602_50323/JPN/2022	Japan	Stool
**OP074133.1**	MAG: Bacteriophage sp. isolate 1697_68134, complete genome	Phage/St/1697_68134/JPN/2022	Japan	Stool
**OP030839.1**	MAG: Bacteriophage sp. isolate 0854_68857, partial genome	Phage/St/0854_68857/JPN/2022	Japan	Stool
**OP072689.1**	MAG: Bacteriophage sp. isolate 3589_105980, partial genome	Phage/St/3589_105980/JPN/2022	Japan	Stool

**OP072302.1**	MAG: Bacteriophage sp. isolate 2414_53161, partial genome	Phage/St/2414_53161/JPN/2022	Japan	Stool
**OP072214.1**	MAG: Bacteriophage sp. isolate 2110_71125, partial genome	Phage/St/2110_71125/JPN/2022	Japan	Stool
**OP031015.1**	MAG: Bacteriophage sp. isolate 1675_92049, partial genome	Phage/St/1675_92049/JPN/2022	Japan	Stool

Table S3. Estimates of Evolutionary Divergence between Sequences of the MCP protein. The red borders refer to the closest distance with current studied sequences.

Species 1	Species 2	Dist
**CrAssphage/WW/2M/SA/2022**	**Phage/St/3057 98020/JPN/2022**	**0.00000000**
**CrAssphage/WW/2M/SA/2022**	**Phage/St/2683 104905/JPN/2022**	**0.00000000**
**CrAssphage/WW/2M/SA/2022**	**Phage/St/4238 99953/JPN/2022**	**0.00000000**
CrAssphage/WW/2M/SA/2022	CrAssphage/WW/7E/SA/2022	0.00281722
**CrAssphage/WW/7E/SA/2022**	**Phage/St/3057 98020/JPN/2022**	**0.00281722**
CrAssphage/WW/2M/SA/2022	Phage/St/3729 82489/JPN/2022	0.00281722
**CrAssphage/WW/12U/SA/2022**	**Phage/St/3729 82489/JPN/2022**	**0.00281722**
CrAssphage/WW/7E/SA/2022	Phage/St/2683 104905/JPN/2022	0.00281722
CrAssphage/WW/7E/SA/2022	Phage/St/4238 99953/JPN/2022	0.00281722
**CrAssphage/WW/2M/SA/2022**	**CrAssphage/WW/12E/SA/2022**	**0.00281728**
**CrAssphage/WW/12E/SA/2022**	**Phage/St/3057 98020/JPN/2022**	**0.00281728**
**CrAssphage/WW/5U/SA/2022**	**Phage/St/3729 82489/JPN/2022**	**0.00281728**
**CrAssphage/WW/12E/SA/2022**	**Phage/St/2683 104905/JPN/2022**	**0.00281728**
**CrAssphage/WW/12E/SA/2022**	**Phage/St/4238 99953/JPN/2022**	**0.00281728**
CrAssphage/WW/5U/SA/2022	CrAssphage/WW/12E/SA/2022	0.00281735
CrAssphage/WW/12E/SA/2022	Phage/St/1876 43632/JPN/2022	0.00281735
CrAssphage/WW/2M/SA/2022	Phage/St/2073 87643/JPN/2023	0.00286957
CrAssphage/WW/2M/SA/2022	Phage/St/3980 139557/JPN/2022	0.00286957
CrAssphage/WW/5U/SA/2022	Phage/St/2938 98355/JPN/2023	0.00287020
CrAssphage/WW/5U/SA/2022	Phage/St/0634 44215/JPN/2022	0.00287020
CrAssphage/WW/5U/SA/2022	Phage/St/0597 88707/JPN/2022	0.00287020
CrAssphage/WW/5U/SA/2022	Phage/St/4174 71253/JPN/2022	0.00287020
CrAssphage/WW/5U/SA/2022	Phage/St/3478 90668/JPN/2022	0.00287020
CrAssphage/WW/5U/SA/2022	Phage/St/2423 107994/JPN/2022	0.00287020
CrAssphage/WW/2M/SA/2022	Phage/St/1876 43632/JPN/2022	0.00287020
CrAssphage/WW/2M/SA/2022	Phage/St/PF-P004 38259/JPN/2023	0.00287020
CrAssphage/WW/12E/SA/2022	Phage/St/1043 114173/JPN/2022	0.00287085
CrAssphage/WW/12E/SA/2022	CrAssphage/HuG/YS1-2 2437/JPN/2020	0.00287085
CrAssphage/WW/12E/SA/2022	CrAssphage/HuG/FA1-2 000172F/JPN/2020	0.00287085
CrAssphage/WW/12E/SA/2022	CrAssphage/HuG/ES ALL 000190F/JPN/2020	0.00287085
CrAssphage/WW/12E/SA/2022	Phage/St/PF-P005 31277/JPN/2023	0.00287085
CrAssphage/WW/12E/SA/2022	Phage/St/0498 70082/JPN/2022	0.00287085
CrAssphage/WW/7E/SA/2022	Phage/St/2073 87643/JPN/2023	0.00585459
CrAssphage/WW/7E/SA/2022	Phage/St/3980 139557/JPN/2022	0.00585459
CrAssphage/WW/12E/SA/2022	Phage/St/2073 87643/JPN/2023	0.00585498
CrAssphage/WW/12E/SA/2022	Phage/St/3980 139557/JPN/2022	0.00585498
CrAssphage/WW/12E/SA/2022	Phage/St/2938 98355/JPN/2023	0.00585538
CrAssphage/WW/12E/SA/2022	Phage/St/0634 44215/JPN/2022	0.00585538
CrAssphage/WW/12E/SA/2022	Phage/St/0597 88707/JPN/2022	0.00585538
CrAssphage/WW/12E/SA/2022	Phage/St/4174 71253/JPN/2022	0.00585538
CrAssphage/WW/12E/SA/2022	Phage/St/3478 90668/JPN/2022	0.00585538
CrAssphage/WW/12E/SA/2022	Phage/St/2423 107994/JPN/2022	0.00585538
CrAssphage/WW/7E/SA/2022	Phage/St/1876 43632/JPN/2022	0.00585538

CrAssphage/WW/2M/SA/2022	Phage/St/1900 63868/JPN/2022	0.00585538
CrAssphage/WW/7E/SA/2022	Phage/St/PF-P004 38259/JPN/2023	0.00585538
CrAssphage/WW/2M/SA/2022	Phage/St/1043 114173/JPN/2022	0.00585579
CrAssphage/WW/12E/SA/2022	Phage/St/PF-P004 38259/JPN/2023	0.00585579
CrAssphage/WW/2M/SA/2022	CrAssphage/HuG/YS1-2 2437/JPN/2020	0.00585579
CrAssphage/WW/2M/SA/2022	CrAssphage/HuG/FA1-2 000172F/JPN/2020	0.00585579
CrAssphage/WW/2M/SA/2022	CrAssphage/HuG/ES ALL 000190F/JPN/2020	0.00585579
CrAssphage/WW/2M/SA/2022	Phage/St/PF-P005 31277/JPN/2023	0.00585579
CrAssphage/WW/2M/SA/2022	Phage/St/0498 70082/JPN/2022	0.00585579
**CrAssphage/WW/4E/SA/2022**	**Phage/St/1043 114173/JPN/2022**	**0.00585620**
CrAssphage/WW/5U/SA/2022	Phage/St/1043 114173/JPN/2022	0.00585620
**CrAssphage/WW/4E/SA/2022**	**CrAssphage/HuG/YS1-2 2437/JPN/2020**	**0.00585620**
CrAssphage/WW/5U/SA/2022	CrAssphage/HuG/YS1-2 2437/JPN/2020	0.00585620
**CrAssphage/WW/4E/SA/2022**	**CrAssphage/HuG/FA1-2 000172F/JPN/2020**	**0.00585620**
CrAssphage/WW/5U/SA/2022	CrAssphage/HuG/FA1-2 000172F/JPN/2020	0.00585620
**CrAssphage/WW/4E/SA/2022**	**CrAssphage/HuG/ES ALL 000190F/JPN/2020**	**0.00585620**
CrAssphage/WW/5U/SA/2022	CrAssphage/HuG/ES ALL 000190F/JPN/2020	0.00585620
**CrAssphage/WW/1E/SA/2022**	**Phage/St/1900 63868/JPN/2022**	**0.00585620**
CrAssphage/WW/4E/SA/2022	Phage/St/PF-P005 31277/JPN/2023	0.00585620
CrAssphage/WW/5U/SA/2022	Phage/St/PF-P005 31277/JPN/2023	0.00585620
CrAssphage/WW/4E/SA/2022	Phage/St/0498 70082/JPN/2022	0.00585620
CrAssphage/WW/5U/SA/2022	Phage/St/0498 70082/JPN/2022	0.00585620
CrAssphage/WW/1E/SA/2022	Phage/St/1043 114173/JPN/2022	0.00585662
CrAssphage/WW/1E/SA/2022	CrAssphage/HuG/YS1-2 2437/JPN/2020	0.00585662
CrAssphage/WW/1E/SA/2022	CrAssphage/HuG/FA1-2 000172F/JPN/2020	0.00585662
CrAssphage/WW/1E/SA/2022	CrAssphage/HuG/ES ALL 000190F/JPN/2020	0.00585662
CrAssphage/WW/1E/SA/2022	Phage/St/PF-P005 31277/JPN/2023	0.00585662
CrAssphage/WW/1E/SA/2022	Phage/St/0498 70082/JPN/2022	0.00585662
**CrAssphage/WW/3E/SA/2022**	**Phage/St/2073 87643/JPN/2023**	**0.00589505**
**CrAssphage/WW/3E/SA/2022**	**Phage/St/3980 139557/JPN/2022**	**0.00589505**
CrAssphage/WW/2M/SA/2022	CrAssphage/WW/3E/SA/2022	0.00589533
CrAssphage/WW/2M/SA/2022	CrAssphage/WW/12U/SA/2022	0.00589533
CrAssphage/WW/3E/SA/2022	Phage/St/3057 98020/JPN/2022	0.00589533
CrAssphage/WW/12U/SA/2022	Phage/St/3057 98020/JPN/2022	0.00589533
CrAssphage/WW/7E/SA/2022	Phage/St/3729 82489/JPN/2022	0.00589533
CrAssphage/WW/3E/SA/2022	Phage/St/2683 104905/JPN/2022	0.00589533
CrAssphage/WW/12U/SA/2022	Phage/St/2683 104905/JPN/2022	0.00589533
CrAssphage/WW/3E/SA/2022	Phage/St/4238 99953/JPN/2022	0.00589533
CrAssphage/WW/12U/SA/2022	Phage/St/4238 99953/JPN/2022	0.00589533
CrAssphage/WW/2M/SA/2022	CrAssphage/WW/5U/SA/2022	0.00589560
CrAssphage/WW/7E/SA/2022	CrAssphage/WW/12E/SA/2022	0.00589560
CrAssphage/WW/5U/SA/2022	CrAssphage/WW/12U/SA/2022	0.00589560
CrAssphage/WW/5U/SA/2022	Phage/St/3057 98020/JPN/2022	0.00589560
CrAssphage/WW/12E/SA/2022	Phage/St/3729 82489/JPN/2022	0.00589560
CrAssphage/WW/5U/SA/2022	Phage/St/2683 104905/JPN/2022	0.00589560
CrAssphage/WW/5U/SA/2022	Phage/St/4238 99953/JPN/2022	0.00589560
CrAssphage/WW/4E/SA/2022	Phage/St/4255 5393/JPN/2023	0.00589588
CrAssphage/WW/4E/SA/2022	Phage/St/3206/JPN/2023	0.00589588

CrAssphage/WW/4E/SA/2022	Phage/St/2775 102739/JPN/2022	0.00589588
CrAssphage/WW/5U/SA/2022	Phage/St/1876 43632/JPN/2022	0.00589588
CrAssphage/WW/3E/SA/2022	CrAssphage/WW/10E/SA/2022	0.00612073
CrAssphage/WW/2M/SA/2022	Phage/St/3519 63130/JPN/2023	0.00612073
CrAssphage/WW/2M/SA/2022	Phage/St/1602 50323/JPN/2022	0.00612073
CrAssphage/WW/1E/SA/2022	CrAssphage/WW/2M/SA/2022	0.00612353
CrAssphage/WW/4E/SA/2022	CrAssphage/WW/5U/SA/2022	0.00612353
CrAssphage/WW/4E/SA/2022	Phage/St/4004 62614/JPN/2023	0.00612353
CrAssphage/WW/1E/SA/2022	Phage/St/3057 98020/JPN/2022	0.00612353
CrAssphage/WW/1E/SA/2022	Phage/St/2683 104905/JPN/2022	0.00612353
CrAssphage/WW/12E/SA/2022	Phage/St/1900 63868/JPN/2022	0.00612353
CrAssphage/WW/1E/SA/2022	Phage/St/4238 99953/JPN/2022	0.00612353
CrAssphage/WW/12U/SA/2022	Phage/St/2073 87643/JPN/2023	0.00911067
CrAssphage/WW/12U/SA/2022	Phage/St/3980 139557/JPN/2022	0.00911067
CrAssphage/WW/2M/SA/2022	Phage/St/2938 98355/JPN/2023	0.00911129
CrAssphage/WW/12U/SA/2022	Phage/St/2938 98355/JPN/2023	0.00911129
CrAssphage/WW/2M/SA/2022	Phage/St/0634 44215/JPN/2022	0.00911129
CrAssphage/WW/12U/SA/2022	Phage/St/0634 44215/JPN/2022	0.00911129
CrAssphage/WW/2M/SA/2022	Phage/St/0597 88707/JPN/2022	0.00911129
CrAssphage/WW/12U/SA/2022	Phage/St/0597 88707/JPN/2022	0.00911129
CrAssphage/WW/2M/SA/2022	Phage/St/4174 71253/JPN/2022	0.00911129
CrAssphage/WW/12U/SA/2022	Phage/St/4174 71253/JPN/2022	0.00911129
CrAssphage/WW/2M/SA/2022	Phage/St/3478 90668/JPN/2022	0.00911129
CrAssphage/WW/12U/SA/2022	Phage/St/3478 90668/JPN/2022	0.00911129
CrAssphage/WW/2M/SA/2022	Phage/St/2423 107994/JPN/2022	0.00911129
CrAssphage/WW/12U/SA/2022	Phage/St/2423 107994/JPN/2022	0.00911129
CrAssphage/WW/3E/SA/2022	Phage/St/3519 63130/JPN/2023	0.00911129
CrAssphage/WW/3E/SA/2022	Phage/St/1602 50323/JPN/2022	0.00911129
CrAssphage/WW/5U/SA/2022	Phage/St/2073 87643/JPN/2023	0.00911129
CrAssphage/WW/5U/SA/2022	Phage/St/3980 139557/JPN/2022	0.00911129
CrAssphage/WW/3E/SA/2022	Phage/St/1876 43632/JPN/2022	0.00911192
CrAssphage/WW/12U/SA/2022	Phage/St/1876 43632/JPN/2022	0.00911192
CrAssphage/WW/3E/SA/2022	Phage/St/PF-P004 38259/JPN/2023	0.00911192
CrAssphage/WW/12U/SA/2022	Phage/St/PF-P004 38259/JPN/2023	0.00911192
CrAssphage/WW/7E/SA/2022	Phage/St/1900 63868/JPN/2022	0.00911192
CrAssphage/WW/7E/SA/2022	Phage/St/1043 114173/JPN/2022	0.00911256
CrAssphage/WW/5U/SA/2022	Phage/St/PF-P004 38259/JPN/2023	0.00911256
CrAssphage/WW/7E/SA/2022	CrAssphage/HuG/YS1-2 2437/JPN/2020	0.00911256
CrAssphage/WW/7E/SA/2022	CrAssphage/HuG/FA1-2 000172F/JPN/2020	0.00911256
CrAssphage/WW/7E/SA/2022	CrAssphage/HuG/ES ALL 000190F/JPN/2020	0.00911256
CrAssphage/WW/7E/SA/2022	Phage/St/PF-P005 31277/JPN/2023	0.00911256
CrAssphage/WW/7E/SA/2022	Phage/St/0498 70082/JPN/2022	0.00911256
CrAssphage/WW/3E/SA/2022	CrAssphage/WW/7E/SA/2022	0.00926260
CrAssphage/WW/7E/SA/2022	CrAssphage/WW/12U/SA/2022	0.00926260
CrAssphage/WW/3E/SA/2022	Phage/St/3729 82489/JPN/2022	0.00926260
CrAssphage/WW/5U/SA/2022	CrAssphage/WW/7E/SA/2022	0.00926326
CrAssphage/WW/3E/SA/2022	CrAssphage/WW/12E/SA/2022	0.00926326
CrAssphage/WW/12E/SA/2022	CrAssphage/WW/12U/SA/2022	0.00926326

CrAssphage/WW/12E/SA/2022	Phage/St/3938 15233/JPN/2023	0.00929717
CrAssphage/WW/7E/SA/2022	Phage/St/3519 63130/JPN/2023	0.00929717
CrAssphage/WW/7E/SA/2022	Phage/St/1602 50323/JPN/2022	0.00929717
CrAssphage/WW/4E/SA/2022	Phage/St/3729 82489/JPN/2022	0.00929871
CrAssphage/WW/12E/SA/2022	Phage/St/3519 63130/JPN/2023	0.00929871
CrAssphage/WW/12E/SA/2022	Phage/St/1602 50323/JPN/2022	0.00929871
CrAssphage/WW/1E/SA/2022	CrAssphage/WW/7E/SA/2022	0.00930027
CrAssphage/WW/4E/SA/2022	CrAssphage/WW/12E/SA/2022	0.00930027
CrAssphage/WW/12E/SA/2022	Phage/St/4255 5393/JPN/2023	0.00930027
**CrAssphage/WW/10E/SA/2022**	**Phage/St/3253 10036/JPN/2023**	**0.00930027**
**CrAssphage/WW/10E/SA/2022**	**Phage/St/2797 3646/JPN/2022**	**0.00930027**
CrAssphage/WW/12E/SA/2022	Phage/St/3206/JPN/2023	0.00930027
CrAssphage/WW/12E/SA/2022	Phage/St/2775 102739/JPN/2022	0.00930027
CrAssphage/WW/3E/SA/2022	Phage/St/3552 133772/JPN/2023	0.00930027
CrAssphage/WW/1E/SA/2022	Phage/St/3729 82489/JPN/2022	0.00930027
CrAssphage/WW/4E/SA/2022	Phage/St/1900 63868/JPN/2022	0.00930027
CrAssphage/WW/5U/SA/2022	Phage/St/1900 63868/JPN/2022	0.00930027
CrAssphage/WW/1E/SA/2022	CrAssphage/WW/12E/SA/2022	0.00930185
CrAssphage/WW/2M/SA/2022	Phage/St/3938 15233/JPN/2023	0.00980249
CrAssphage/WW/2M/SA/2022	Phage/St/4255 5393/JPN/2023	0.00980929
CrAssphage/WW/4E/SA/2022	Phage/St/2938 98355/JPN/2023	0.00980929
CrAssphage/WW/4E/SA/2022	Phage/St/0634 44215/JPN/2022	0.00980929
CrAssphage/WW/4E/SA/2022	Phage/St/0597 88707/JPN/2022	0.00980929
CrAssphage/WW/4E/SA/2022	Phage/St/4174 71253/JPN/2022	0.00980929
CrAssphage/WW/4E/SA/2022	Phage/St/3478 90668/JPN/2022	0.00980929
CrAssphage/WW/4E/SA/2022	Phage/St/2423 107994/JPN/2022	0.00980929
CrAssphage/WW/2M/SA/2022	Phage/St/3206/JPN/2023	0.00980929
CrAssphage/WW/2M/SA/2022	Phage/St/2775 102739/JPN/2022	0.00980929
CrAssphage/WW/1E/SA/2022	Phage/St/2073 87643/JPN/2023	0.00980929
CrAssphage/WW/1E/SA/2022	Phage/St/3980 139557/JPN/2022	0.00980929
CrAssphage/WW/1E/SA/2022	Phage/St/4255 5393/JPN/2023	0.00981626
CrAssphage/WW/1E/SA/2022	Phage/St/3206/JPN/2023	0.00981626
CrAssphage/WW/1E/SA/2022	Phage/St/2775 102739/JPN/2022	0.00981626
CrAssphage/WW/1E/SA/2022	Phage/St/1876 43632/JPN/2022	0.00981626
CrAssphage/WW/1E/SA/2022	Phage/St/PF-P004 38259/JPN/2023	0.00981626
CrAssphage/WW/7E/SA/2022	Phage/St/2938 98355/JPN/2023	0.01266775
CrAssphage/WW/7E/SA/2022	Phage/St/0634 44215/JPN/2022	0.01266775
CrAssphage/WW/7E/SA/2022	Phage/St/0597 88707/JPN/2022	0.01266775
CrAssphage/WW/7E/SA/2022	Phage/St/4174 71253/JPN/2022	0.01266775
CrAssphage/WW/7E/SA/2022	Phage/St/3478 90668/JPN/2022	0.01266775
CrAssphage/WW/7E/SA/2022	Phage/St/2423 107994/JPN/2022	0.01266775
CrAssphage/WW/3E/SA/2022	Phage/St/1900 63868/JPN/2022	0.01266879
CrAssphage/WW/12U/SA/2022	Phage/St/1900 63868/JPN/2022	0.01266879
CrAssphage/WW/3E/SA/2022	Phage/St/1043 114173/JPN/2022	0.01266983
CrAssphage/WW/12U/SA/2022	Phage/St/1043 114173/JPN/2022	0.01266983
CrAssphage/WW/3E/SA/2022	CrAssphage/HuG/YS1-2 2437/JPN/2020	0.01266983
CrAssphage/WW/12U/SA/2022	CrAssphage/HuG/YS1-2 2437/JPN/2020	0.01266983
CrAssphage/WW/3E/SA/2022	CrAssphage/HuG/FA1-2 000172F/JPN/2020	0.01266983

CrAssphage/WW/12U/SA/2022	CrAssphage/HuG/FA1-2 000172F/JPN/2020	0.01266983
CrAssphage/WW/3E/SA/2022	CrAssphage/HuG/ES ALL 000190F/JPN/2020	0.01266983
CrAssphage/WW/12U/SA/2022	CrAssphage/HuG/ES ALL 000190F/JPN/2020	0.01266983
CrAssphage/WW/3E/SA/2022	Phage/St/PF-P005 31277/JPN/2023	0.01266983
CrAssphage/WW/12U/SA/2022	Phage/St/PF-P005 31277/JPN/2023	0.01266983
CrAssphage/WW/3E/SA/2022	Phage/St/0498 70082/JPN/2022	0.01266983
CrAssphage/WW/12U/SA/2022	Phage/St/0498 70082/JPN/2022	0.01266983
CrAssphage/WW/3E/SA/2022	Phage/St/3938 15233/JPN/2023	0.01275483
CrAssphage/WW/10E/SA/2022	Phage/St/2073 87643/JPN/2023	0.01275483
CrAssphage/WW/10E/SA/2022	Phage/St/3980 139557/JPN/2022	0.01275483
CrAssphage/WW/2M/SA/2022	CrAssphage/WW/10E/SA/2022	0.01275665
CrAssphage/WW/5U/SA/2022	Phage/St/3938 15233/JPN/2023	0.01275665
CrAssphage/WW/12U/SA/2022	Phage/St/3519 63130/JPN/2023	0.01275665
CrAssphage/WW/12U/SA/2022	Phage/St/1602 50323/JPN/2022	0.01275665
CrAssphage/WW/10E/SA/2022	Phage/St/3057 98020/JPN/2022	0.01275665
CrAssphage/WW/10E/SA/2022	Phage/St/2683 104905/JPN/2022	0.01275665
CrAssphage/WW/10E/SA/2022	Phage/St/4238 99953/JPN/2022	0.01275665
CrAssphage/WW/2M/SA/2022	CrAssphage/WW/4E/SA/2022	0.01275849
CrAssphage/WW/4E/SA/2022	Phage/St/3057 98020/JPN/2022	0.01275849
CrAssphage/WW/4E/SA/2022	Phage/St/2683 104905/JPN/2022	0.01275849
CrAssphage/WW/4E/SA/2022	Phage/St/4238 99953/JPN/2022	0.01275849
CrAssphage/WW/4E/SA/2022	CrAssphage/WW/12U/SA/2022	0.01275849
CrAssphage/WW/5U/SA/2022	Phage/St/3519 63130/JPN/2023	0.01275849
CrAssphage/WW/5U/SA/2022	Phage/St/1602 50323/JPN/2022	0.01275849
CrAssphage/WW/1E/SA/2022	CrAssphage/WW/3E/SA/2022	0.01276035
CrAssphage/WW/1E/SA/2022	CrAssphage/WW/12U/SA/2022	0.01276035
CrAssphage/WW/5U/SA/2022	Phage/St/4255 5393/JPN/2023	0.01276035
CrAssphage/WW/5U/SA/2022	Phage/St/3206/JPN/2023	0.01276035
CrAssphage/WW/5U/SA/2022	Phage/St/2775 102739/JPN/2022	0.01276035
CrAssphage/WW/4E/SA/2022	Phage/St/1876 43632/JPN/2022	0.01276035
CrAssphage/WW/1E/SA/2022	CrAssphage/WW/4E/SA/2022	0.01276224
CrAssphage/WW/1E/SA/2022	CrAssphage/WW/5U/SA/2022	0.01276224
CrAssphage/WW/3E/SA/2022	CrAssphage/WW/12U/SA/2022	0.01295079
CrAssphage/WW/3E/SA/2022	Phage/St/0597 88707/JPN/2022	0.01295079
CrAssphage/WW/3E/SA/2022	CrAssphage/WW/5U/SA/2022	0.01295203
CrAssphage/WW/7E/SA/2022	Phage/St/3938 15233/JPN/2023	0.01319690
CrAssphage/WW/7E/SA/2022	Phage/St/4255 5393/JPN/2023	0.01320424
CrAssphage/WW/7E/SA/2022	Phage/St/3206/JPN/2023	0.01320424
CrAssphage/WW/7E/SA/2022	Phage/St/2775 102739/JPN/2022	0.01320424
CrAssphage/WW/5U/SA/2022	Phage/St/4004 62614/JPN/2023	0.01399986
CrAssphage/WW/1E/SA/2022	Phage/St/3519 63130/JPN/2023	0.01399986
CrAssphage/WW/1E/SA/2022	Phage/St/1602 50323/JPN/2022	0.01399986
CrAssphage/WW/7E/SA/2022	CrAssphage/WW/10E/SA/2022	0.01652888
CrAssphage/WW/10E/SA/2022	Phage/St/3729 82489/JPN/2022	0.01652888
CrAssphage/WW/4E/SA/2022	CrAssphage/WW/7E/SA/2022	0.01653119
CrAssphage/WW/10E/SA/2022	CrAssphage/WW/12E/SA/2022	0.01653119
CrAssphage/WW/2M/SA/2022	Phage/St/3552 133772/JPN/2023	0.01653354
CrAssphage/WW/3E/SA/2022	Phage/St/2938 98355/JPN/2023	0.01655682

CrAssphage/WW/3E/SA/2022	Phage/St/0634 44215/JPN/2022	0.01655682
CrAssphage/WW/3E/SA/2022	Phage/St/4174 71253/JPN/2022	0.01655682
CrAssphage/WW/3E/SA/2022	Phage/St/3478 90668/JPN/2022	0.01655682
CrAssphage/WW/3E/SA/2022	Phage/St/2423 107994/JPN/2022	0.01655682
CrAssphage/WW/12E/SA/2022	Phage/St/3552 133772/JPN/2023	0.01656179
CrAssphage/WW/12U/SA/2022	Phage/St/3938 15233/JPN/2023	0.01688776
CrAssphage/WW/10E/SA/2022	Phage/St/3519 63130/JPN/2023	0.01689184
CrAssphage/WW/10E/SA/2022	Phage/St/1602 50323/JPN/2022	0.01689184
CrAssphage/WW/4E/SA/2022	Phage/St/2073 87643/JPN/2023	0.01689184
CrAssphage/WW/4E/SA/2022	Phage/St/3980 139557/JPN/2022	0.01689184
CrAssphage/WW/3E/SA/2022	Phage/St/4255 5393/JPN/2023	0.01689597
CrAssphage/WW/12U/SA/2022	Phage/St/4255 5393/JPN/2023	0.01689597
CrAssphage/WW/3E/SA/2022	Phage/St/3206/JPN/2023	0.01689597
CrAssphage/WW/12U/SA/2022	Phage/St/3206/JPN/2023	0.01689597
CrAssphage/WW/3E/SA/2022	Phage/St/2775 102739/JPN/2022	0.01689597
CrAssphage/WW/12U/SA/2022	Phage/St/2775 102739/JPN/2022	0.01689597
CrAssphage/WW/10E/SA/2022	Phage/St/1876 43632/JPN/2022	0.01689597
CrAssphage/WW/10E/SA/2022	Phage/St/PF-P004 38259/JPN/2023	0.01689597
CrAssphage/WW/1E/SA/2022	Phage/St/2938 98355/JPN/2023	0.01690015
CrAssphage/WW/1E/SA/2022	Phage/St/0634 44215/JPN/2022	0.01690015
CrAssphage/WW/1E/SA/2022	Phage/St/0597 88707/JPN/2022	0.01690015
CrAssphage/WW/1E/SA/2022	Phage/St/4174 71253/JPN/2022	0.01690015
CrAssphage/WW/1E/SA/2022	Phage/St/3478 90668/JPN/2022	0.01690015
CrAssphage/WW/1E/SA/2022	Phage/St/2423 107994/JPN/2022	0.01690015
CrAssphage/WW/4E/SA/2022	Phage/St/PF-P004 38259/JPN/2023	0.01690015
CrAssphage/WW/3E/SA/2022	Phage/St/3253 10036/JPN/2023	0.01764462
CrAssphage/WW/3E/SA/2022	Phage/St/2797 3646/JPN/2022	0.01764462
CrAssphage/WW/12E/SA/2022	Phage/St/4004 62614/JPN/2023	0.01764462
CrAssphage/WW/10E/SA/2022	Phage/St/3552 133772/JPN/2023	0.01764462
CrAssphage/WW/1E/SA/2022	Phage/St/3938 15233/JPN/2023	0.01874413
CrAssphage/WW/10E/SA/2022	CrAssphage/WW/12U/SA/2022	0.02064712
CrAssphage/WW/10E/SA/2022	Phage/St/0597 88707/JPN/2022	0.02064712
CrAssphage/WW/3E/SA/2022	CrAssphage/WW/4E/SA/2022	0.02065013
CrAssphage/WW/5U/SA/2022	CrAssphage/WW/10E/SA/2022	0.02065013
CrAssphage/WW/7E/SA/2022	Phage/St/3552 133772/JPN/2023	0.02065317
CrAssphage/WW/5U/SA/2022	Phage/St/3552 133772/JPN/2023	0.02082245
CrAssphage/WW/10E/SA/2022	Phage/St/1900 63868/JPN/2022	0.02091510
CrAssphage/WW/10E/SA/2022	Phage/St/1043 114173/JPN/2022	0.02091991
CrAssphage/WW/10E/SA/2022	CrAssphage/HuG/YS1-2 2437/JPN/2020	0.02091991
CrAssphage/WW/10E/SA/2022	CrAssphage/HuG/FA1-2 000172F/JPN/2020	0.02091991
CrAssphage/WW/10E/SA/2022	CrAssphage/HuG/ES ALL 000190F/JPN/2020	0.02091991
CrAssphage/WW/10E/SA/2022	Phage/St/PF-P005 31277/JPN/2023	0.02091991
CrAssphage/WW/10E/SA/2022	Phage/St/0498 70082/JPN/2022	0.02091991
CrAssphage/WW/10E/SA/2022	Phage/St/3938 15233/JPN/2023	0.02157856
CrAssphage/WW/4E/SA/2022	Phage/St/3938 15233/JPN/2023	0.02158625
CrAssphage/WW/2M/SA/2022	Phage/St/4004 62614/JPN/2023	0.02159404
CrAssphage/WW/12U/SA/2022	Phage/St/4004 62614/JPN/2023	0.02159404
CrAssphage/WW/4E/SA/2022	Phage/St/3519 63130/JPN/2023	0.02159404

CrAssphage/WW/4E/SA/2022	Phage/St/1602 50323/JPN/2022	0.02159404
CrAssphage/WW/1E/SA/2022	CrAssphage/WW/10E/SA/2022	0.02160194
CrAssphage/WW/1E/SA/2022	Phage/St/4004 62614/JPN/2023	0.02160993
CrAssphage/WW/12U/SA/2022	Phage/St/3552 133772/JPN/2023	0.02515661
CrAssphage/WW/10E/SA/2022	Phage/St/2938 98355/JPN/2023	0.02529028
CrAssphage/WW/10E/SA/2022	Phage/St/0634 44215/JPN/2022	0.02529028
CrAssphage/WW/10E/SA/2022	Phage/St/4174 71253/JPN/2022	0.02529028
CrAssphage/WW/10E/SA/2022	Phage/St/3478 90668/JPN/2022	0.02529028
CrAssphage/WW/10E/SA/2022	Phage/St/2423 107994/JPN/2022	0.02529028
CrAssphage/WW/12E/SA/2022	Phage/St/3253 10036/JPN/2023	0.02530726
CrAssphage/WW/12E/SA/2022	Phage/St/2797 3646/JPN/2022	0.02530726
CrAssphage/WW/7E/SA/2022	Phage/St/4004 62614/JPN/2023	0.02589473
CrAssphage/WW/2M/SA/2022	Phage/St/3253 10036/JPN/2023	0.02590348
CrAssphage/WW/2M/SA/2022	Phage/St/2797 3646/JPN/2022	0.02590348
CrAssphage/WW/1E/SA/2022	Phage/St/3552 133772/JPN/2023	0.02592134
CrAssphage/WW/10E/SA/2022	Phage/St/4255 5393/JPN/2023	0.02693279
CrAssphage/WW/10E/SA/2022	Phage/St/3206/JPN/2023	0.02693279
CrAssphage/WW/10E/SA/2022	Phage/St/2775 102739/JPN/2022	0.02693279
CrAssphage/WW/5U/SA/2022	Phage/St/3253 10036/JPN/2023	0.03009026
CrAssphage/WW/5U/SA/2022	Phage/St/2797 3646/JPN/2022	0.03009026
CrAssphage/WW/4E/SA/2022	Phage/St/3552 133772/JPN/2023	0.03009026
CrAssphage/WW/4E/SA/2022	CrAssphage/WW/10E/SA/2022	0.03057479
CrAssphage/WW/3E/SA/2022	Phage/St/4004 62614/JPN/2023	0.03057479
CrAssphage/WW/7E/SA/2022	Phage/St/3253 10036/JPN/2023	0.03058465
CrAssphage/WW/7E/SA/2022	Phage/St/2797 3646/JPN/2022	0.03058465
CrAssphage/WW/12U/SA/2022	Phage/St/3253 10036/JPN/2023	0.03568498
CrAssphage/WW/12U/SA/2022	Phage/St/2797 3646/JPN/2022	0.03568498
CrAssphage/WW/1E/SA/2022	Phage/St/3253 10036/JPN/2023	0.03806436
CrAssphage/WW/1E/SA/2022	Phage/St/2797 3646/JPN/2022	0.03806436
CrAssphage/WW/4E/SA/2022	Phage/St/3253 10036/JPN/2023	0.04207676
CrAssphage/WW/4E/SA/2022	Phage/St/2797 3646/JPN/2022	0.04207676
CrAssphage/WW/10E/SA/2022	Phage/St/4004 62614/JPN/2023	0.04340990

Table S4. Best fitting nucleotide substitution model for MP-PBD gene

Model	#Param	BIC	AICc	lnL	Invariant	Gamma	R	Freq A	Freq T	Freq C	Freq G
T92+G+I	49	1334.201277	1012.468065	-456.7717684	0.838338615	0.44	0.65195311	0.354112	0.354112	0.1458879	0.1458879
T92+G	48	1340.711908	1025.526399	-464.3195097	n/a	0.05	0.55932358	0.354112	0.354112	0.1458879	0.1458879
HKY+G+I	51	1351.241897	1016.415586	-456.7072265	0.836512893	0.43	0.65856137	0.343178	0.365047	0.1295327	0.162243
HKY+G	50	1357.762613	1029.482467	-464.2600106	n/a	0.05	0.55979417	0.343178	0.365047	0.1295327	0.162243
TN93+G+I	52	1358.507589	1017.135883	-456.0476469	0.846870616	0.47	0.67140602	0.343178	0.365047	0.1295327	0.162243
TN93+G	51	1365.476248	1030.649937	-463.8244023	n/a	0.05	0.56796827	0.343178	0.365047	0.1295327	0.162243
JC+G+I	47	1367.33528	1058.698243	-481.9236218	0.596064703	0.06	0.5	0.25	0.25	0.25	0.25
T92+I	48	1368.758165	1053.572657	-478.3426386	0.466367713	n/a	0.53397504	0.354112	0.354112	0.1458879	0.1458879
JC+G	46	1371.161423	1069.073626	-488.1291191	n/a	0.05	0.5	0.25	0.25	0.25	0.25
T92	47	1376.730404	1068.093368	-486.621184	n/a	n/a	0.5270582	0.354112	0.354112	0.1458879	0.1458879
K2+G+I	48	1382.328404	1067.142895	-485.1277578	0.590476081	0.06	1.34255137	0.25	0.25	0.25	0.25
GTR+G+I	55	1382.397889	1021.394619	-455.1155187	0.834437329	0.43	0.56783784	0.343178	0.365047	0.1295327	0.162243
K2+G	47	1384.559304	1075.922267	-490.5356336	n/a	0.05	1.25486816	0.25	0.25	0.25	0.25
HKY+I	50	1385.809029	1057.528883	-478.2832186	0.466367713	n/a	0.53416307	0.343178	0.365047	0.1295327	0.162243
GTR+G	54	1386.818782	1032.358596	-461.6183915	n/a	0.05	0.53912503	0.343178	0.365047	0.1295327	0.162243
TN93+I	51	1393.578303	1058.751992	-477.8754295	0.466367713	n/a	0.53578368	0.343178	0.365047	0.1295327	0.162243
HKY	49	1393.782023	1072.048811	-486.5621413	n/a	n/a	0.5271575	0.343178	0.365047	0.1295327	0.162243
JC+I	46	1398.569451	1096.481654	-501.8331331	0.466367713	n/a	0.5	0.25	0.25	0.25	0.25
TN93	50	1401.588101	1073.307954	-486.1727543	n/a	n/a	0.5266347	0.343178	0.365047	0.1295327	0.162243
JC	45	1406.583249	1111.045459	-510.1324581	n/a	n/a	0.5	0.25	0.25	0.25	0.25
K2+I	47	1411.251736	1102.614699	-503.8818496	0.466367713	n/a	1.17778635	0.25	0.25	0.25	0.25
GTR+I	54	1416.218917	1061.75873	-476.3184586	0.466367713	n/a	0.46692076	0.343178	0.365047	0.1295327	0.162243
K2	46	1419.532483	1117.444686	-512.3146494	n/a	n/a	1.17036486	0.25	0.25	0.25	0.25
GTR	53	1424.105896	1076.189565	-484.5543745	n/a	n/a	0.464803	0.343178	0.365047	0.1295327	0.162243

Models with the lowest BIC scores (Bayesian Information Criterion) are considered to describe the substitution pattern the best. For each model, AICc value (Akaike Information Criterion, corrected), Maximum Likelihood value (lnL), and the number of parameters (including branch lengths) are also presented. Non-uniformity of evolutionary rates among sites may be modeled by using a discrete Gamma distribution (+G) with 5 rate categories and by assuming that a certain fraction of sites is evolut ionarily invariable (+I). Whenever applicable, estimates of gamma shape parameter and/or the estimated fraction of invariant sites are shown. (Kumar *et al*., 2018)

*Abbreviations: TR: General Time Reversible; HKY: Hasegawa-Kishino-Yano; TN93: Tamura-Nei; T92: Tamura 3-parameter; K2: Kimura 2-parameter; JC: Jukes-Cantor

Table S5. Sequences used for phylogenetic analysis of CrAssphage MP-PBD gene.

Accession number	Sequence nomination	Abbreviated name used for phylogenetic tree	Country	Source
**OP075001.1**	MAG: Bacteriophage sp. Isolate 2900_14151, partial genome	Phage/St/2900_14151/JPN/2023	Japan	Stool
**OP072767.1**	MAG: Bacteriophage sp. isolate 3798_1224, partial genome	Phage/St/3798_1224/JPN/2022	Japan	Stool
**OP075729.1**	MAG: Bacteriophage sp. isolate 3695_32156, complete genome	Phage/St/3695_32156/JPN/2023	Japan	Stool
**OP074980.1**	MAG: Bacteriophage sp. isolate 2879_32956, partial genome	Phage/St/2879_32956/JPN/2023	Japan	Stool
**OP072609.1**	MAG: Bacteriophage sp. isolate 3389_74325, partial genome	Phage/St/3389_74325/JPN/2022	Japan	Stool
**OP072511.1**	MAG: Bacteriophage sp. isolate 3067_17778, partial genome	Phage/St/3067_17778/JPN/2022	Japan	Stool
**OP075953.1**	MAG: Bacteriophage sp. isolate 3955_36540, partial genome	Phage/St/3955_36540/JPN/2023	Japan	Stool
**OP075799.1**	MAG: Bacteriophage sp. isolate 3786_84692, partial genome	Phage/St/3786_84692/JPN/2023	Japan	Stool
**OP075580.1**	MAG: Bacteriophage sp. isolate 3522_63963, partial genome	Phage/St/3522_63963/JPN/2023	Japan	Stool
**OP075087.1**	MAG: Bacteriophage sp. Isolate 2980_37109, partial genome	Phage/St/2980_37109/JPN/2023	Japan	Stool
**OP030946.1**	MAG: Bacteriophage sp. isolate 1405_61638, partial genome	Phage/St/1405_61638/JPN/2022	Japan	Stool
**OP075443.1**	MAG: Bacteriophage sp. isolate 3388_29408, partial genome	Phage/St/3388_29408/JPN/2023	Japan	Stool
**OP074980.1**	MAG: Bacteriophage sp. isolate 2879_32956, partial genome	Phage/St/2879_32956/JPN/2023	Japan	Stool

**OP074775.1**	MAG: Bacteriophage sp. isolate 2650_35736, complete genome	Phage/St/2650_35736/JPN/2022	Japan	Stool
**OP073572.1**	MAG: Bacteriophage sp. isolate 0937_15216, partial genome	Phage/St/0937_15216/JPN/2022	Japan	Stool
**OP072840.1**	MAG: Bacteriophage sp. isolate 4078_53722, partial genome	Phage/St/4078_53722/JPN/2022	Japan	Stool
**OP072890.1**	MAG: Bacteriophage sp. isolate 4268_83826, complete genome	Phage/St/4268_83826/JPN/2022	Japan	Stool
**OP072218.1**	MAG: Bacteriophage sp. isolate 2121_105414, partial genome	Phage/St/2121_105414/JPN/2022	Japan	Stool
**OP031107.1**	MAG: Bacteriophage sp. isolate 2023_91933, partial genome	Phage/St/2023_91933/JPN/2022	Japan	Stool
**OP030832.1**	MAG: Bacteriophage sp. isolate 0810_75712, partial genome	Phage/St/0810_75712/JPN/2022	Japan	Stool
**OP076428.1**	MAG: Bacteriophage sp. isolate 2025_86962, partial genome	Phage/St/2025_86962/JPN/2023	Japan	Stool
**OP073190.1**	MAG: Bacteriophage sp. isolate 0377_101794, complete genome	Phage/St/0377_101794/JPN/2022	Japan	Stool
**OP030869.1**	MAG: Bacteriophage sp. isolate 1009_62914, complete genome	Phage/St/1009_62914/JPN/2022	Japan	Stool
**MK415410.1**	MAG: CrAssphage YS1-2_2437, complete genome	CrAssphage/HuG/YS1-2_2437/JPN/2020	Japan	human gut
**NC_067194.1**	MAG: Carjivirus communis, complete genome	CrAssphage/St/Carjivirus communis/USA/2023	USA	Stool
**OP075978.1**	MAG: Bacteriophage sp. isolate 3989_33300, partial genome	Phage/St/3989_33300/JPN/2023	Japan	Stool
**OP075780.1**	MAG: Bacteriophage sp. isolate 3764_67264, partial genome	Phage/St/3764_67264/JPN/2023	Japan	Stool
**OP075946.1**	MAG: Bacteriophage sp. isolate 3947_59002, complete genome	Phage/St/3947_59002/JPN/2023	Japan	Stool
**OP075632.1**	MAG: Bacteriophage sp. isolate 3575_41778, partial genome	Phage/St/3575_41778/JPN/2023	Japan	Stool
**OP075506.1**	MAG: Bacteriophage sp. isolate 3451_51812, partial genome	Phage/St/3451_51812/JPN/2023	Japan	Stool

**OP075975.1**	MAG: Bacteriophage sp. isolate 3986_25839, partial genome	Phage/St/3986_25839/JPN/2023	Japan	Stool
**OP075948.1**	MAG: Bacteriophage sp. isolate 3950_21566, complete genome	Phage/St/3950_21566/JPN/2023	Japan	Stool
**OP075612.1**	MAG: Bacteriophage sp. isolate 3556_60220, complete genome	Phage/St/3556_60220/JPN/2023	Japan	Stool
**OP075036.1**	MAG: Bacteriophage sp. isolate 2929_39558, partial genome	Phage/St/2929_39558/JPN/2023	Japan	Stool
**OP074964.1**	MAG: Bacteriophage sp. isolate 2861_38114, partial genome	Phage/St/2861_38114/JPN/2023	Japan	Stool
**OP075180.1**	MAG: Human gut phage 3075_79973 isolate 3075, complete genome	Phage/St/3075/JPN/2023	Japan	Stool
**OP076487.1**	MAG: Bacteriophage sp. isolate 2087_16664, partial genome	Phage/St/2087_16664/JPN/2023	Japan	Stool
**OP076277.1**	MAG: Bacteriophage sp. isolate PF-P005_31277, partial genome	Phage/St/PF-P005_31277/JPN/2023	Japan	Stool
**OP072921.1**	MAG: Bacteriophage sp. isolate PF-HC115_125715, partial genome	Phage/St/PF-HC115_125715/JPN/2022	Japan	Stool
**OP072588.1**	MAG: Bacteriophage sp. isolate 3316_61037, partial genome	Phage/St/3316_61037/JPN/2022	Japan	Stool

Table S6. Estimates of Evolutionary Divergence between Sequences of the MP-PBD protein. The red borders refer to the closest distance with current studied sequences.

Species 1	Species 2	Dist
**CrAssphage/WW/88U/SA/2022**	**Phage/St/3388 29408/JPN/2023**	**0.01002303**
**CrAssphage/WW/88U/SA/2022**	**Phage/St/0937 15216/JPN/2022**	**0.01002303**
**CrAssphage/WW/88U/SA/2022**	**Phage/St/2121 105414/JPN/2022**	**0.01002303**
CrAssphage/WW/111E/SA/2022	Phage/St/0377 101794/JPN/2022	0.01003723
**CrAssphage/WW/88U/SA/2022**	**Phage/St/4268 83826/JPN/2022**	**0.01008693**
**CrAssphage/WW/88U/SA/2022**	**Phage/St/2879 32956/JPN/2023**	**0.01008693**
**CrAssphage/WW/77U/SA/2022**	**CrAssphage/WW/112E/SA/2022**	**0.01071937**
**CrAssphage/WW/111E/SA/2022**	**CrAssphage/HuG/YS1-2 2437/JPN/2020**	**0.01564090**
**CrAssphage/WW/111E/SA/2022**	**Phage/St/3947 59002/JPN/2023**	**0.01564090**
**CrAssphage/WW/111E/SA/2022**	**Phage/St/3451 51812/JPN/2023**	**0.01564090**
**CrAssphage/WW/111E/SA/2022**	**Phage/St/3388 29408/JPN/2023**	**0.01564090**
**CrAssphage/WW/111E/SA/2022**	**Phage/St/0937 15216/JPN/2022**	**0.01564090**
**CrAssphage/WW/111E/SA/2022**	**Phage/St/2121 105414/JPN/2022**	**0.01564090**
CrAssphage/WW/111E/SA/2022	Phage/St/3575 41778/JPN/2023	0.01564452
CrAssphage/WW/77U/SA/2022	Phage/St/2900 14151/JPN/2023	0.01572026
CrAssphage/WW/88U/SA/2022	Phage/St/2900 14151/JPN/2023	0.01572390
CrAssphage/WW/111E/SA/2022	Phage/St/4268 83826/JPN/2022	0.01596326
CrAssphage/WW/111E/SA/2022	Phage/St/2879 32956/JPN/2023	0.01596326
CrAssphage/WW/88U/SA/2022	CrAssphage/WW/111E/SA/2022	0.01605626
CrAssphage/WW/88U/SA/2022	Phage/St/0377 101794/JPN/2022	0.01605969
**CrAssphage/WW/112E/SA/2022**	**Phage/St/3986 25839/JPN/2023**	**0.02196325**
**CrAssphage/WW/112E/SA/2022**	**Phage/St/3950 21566/JPN/2023**	**0.02196325**
**CrAssphage/WW/112E/SA/2022**	**Phage/St/2929 39558/JPN/2023**	**0.02196325**
**CrAssphage/WW/112E/SA/2022**	**Phage/St/2861 38114/JPN/2023**	**0.02196325**
**CrAssphage/WW/112E/SA/2022**	**Phage/St/3075/JPN/2023**	**0.02196325**
**CrAssphage/WW/112E/SA/2022**	**Phage/St/2087 16664/JPN/2023**	**0.02196325**
**CrAssphage/WW/112E/SA/2022**	**Phage/St/PF-P005 31277/JPN/2023**	**0.02196325**
**CrAssphage/WW/112E/SA/2022**	**Phage/St/3316 61037/JPN/2022**	**0.02196325**
CrAssphage/WW/88U/SA/2022	Phage/St/3575 41778/JPN/2023	0.02210874
CrAssphage/WW/77U/SA/2022	Phage/St/2879 32956/JPN/2023	0.02241539
CrAssphage/WW/77U/SA/2022	CrAssphage/WW/88U/SA/2022	0.02242730
CrAssphage/WW/112E/SA/2022	Phage/St/3955 36540/JPN/2023_{outgroup}	0.02910606
CrAssphage/WW/77U/SA/2022	CrAssphage/WW/111E/SA/2022	0.02929428
CrAssphage/WW/88U/SA/2022	CrAssphage/HuG/YS1-2 2437/JPN/2020	0.02929428
CrAssphage/WW/88U/SA/2022	Phage/St/3947 59002/JPN/2023	0.02929428
CrAssphage/WW/88U/SA/2022	Phage/St/3451 51812/JPN/2023	0.02929428
CrAssphage/WW/77U/SA/2022	Phage/St/3955 36540/JPN/2023_{outgroup}	0.02929428
CrAssphage/WW/112E/SA/2022	Phage/St/2900 14151/JPN/2023	0.03012728
CrAssphage/WW/88U/SA/2022	Phage/St/3955 36540/JPN/2023_{outgroup}	0.03258890
CrAssphage/WW/77U/SA/2022	Phage/St/4268 83826/JPN/2022	0.03694566
CrAssphage/WW/111E/SA/2022	Phage/St/2900 14151/JPN/2023	0.03699854

CrAssphage/WW/77U/SA/2022	Phage/St/3986 25839/JPN/2023	0.03779555
CrAssphage/WW/77U/SA/2022	Phage/St/3950 21566/JPN/2023	0.03779555
CrAssphage/WW/77U/SA/2022	Phage/St/2929 39558/JPN/2023	0.03779555
CrAssphage/WW/77U/SA/2022	Phage/St/2861 38114/JPN/2023	0.03779555
CrAssphage/WW/77U/SA/2022	Phage/St/3075/JPN/2023	0.03779555
CrAssphage/WW/77U/SA/2022	Phage/St/2087 16664/JPN/2023	0.03779555
CrAssphage/WW/77U/SA/2022	Phage/St/PF-P005 31277/JPN/2023	0.03779555
CrAssphage/WW/77U/SA/2022	Phage/St/3316 61037/JPN/2022	0.03779555
CrAssphage/WW/77U/SA/2022	Phage/St/3388 29408/JPN/2023	0.03782728
CrAssphage/WW/77U/SA/2022	Phage/St/0937 15216/JPN/2022	0.03782728
CrAssphage/WW/77U/SA/2022	Phage/St/2121 105414/JPN/2022	0.03782728
CrAssphage/WW/112E/SA/2022	Phage/St/2879 32956/JPN/2023	0.03952147
CrAssphage/WW/111E/SA/2022	Phage/St/3955 36540/JPN/2023_{outgroup}	0.03962923
CrAssphage/WW/88U/SA/2022	CrAssphage/WW/112E/SA/2022	0.03990813
CrAssphage/WW/77U/SA/2022	Phage/St/0377 101794/JPN/2022	0.04557413
CrAssphage/WW/111E/SA/2022	CrAssphage/WW/112E/SA/2022	0.04767971
CrAssphage/WW/77U/SA/2022	CrAssphage/HuG/YS1-2 2437/JPN/2020	0.04801986
CrAssphage/WW/77U/SA/2022	Phage/St/3947 59002/JPN/2023	0.04801986
CrAssphage/WW/77U/SA/2022	Phage/St/3451 51812/JPN/2023	0.04801986
CrAssphage/WW/111E/SA/2022	Phage/St/3986 25839/JPN/2023	0.05068257
CrAssphage/WW/111E/SA/2022	Phage/St/3950 21566/JPN/2023	0.05068257
CrAssphage/WW/111E/SA/2022	Phage/St/2929 39558/JPN/2023	0.05068257
CrAssphage/WW/111E/SA/2022	Phage/St/2861 38114/JPN/2023	0.05068257
CrAssphage/WW/111E/SA/2022	Phage/St/2087 16664/JPN/2023	0.05068257
CrAssphage/WW/111E/SA/2022	Phage/St/PF-P005 31277/JPN/2023	0.05068257
CrAssphage/WW/111E/SA/2022	Phage/St/3316 61037/JPN/2022	0.05068257
CrAssphage/WW/112E/SA/2022	Phage/St/0377 101794/JPN/2022	0.05506142
CrAssphage/WW/77U/SA/2022	Phage/St/3575 41778/JPN/2023	0.05544475
CrAssphage/WW/112E/SA/2022	Phage/St/4268 83826/JPN/2022	0.05661275
CrAssphage/WW/112E/SA/2022	CrAssphage/HuG/YS1-2 2437/JPN/2020	0.05962600
CrAssphage/WW/112E/SA/2022	Phage/St/3947 59002/JPN/2023	0.05962600
CrAssphage/WW/112E/SA/2022	Phage/St/3451 51812/JPN/2023	0.05962600
CrAssphage/WW/112E/SA/2022	Phage/St/3388 29408/JPN/2023	0.05962600
CrAssphage/WW/112E/SA/2022	Phage/St/0937 15216/JPN/2022	0.05962600
CrAssphage/WW/112E/SA/2022	Phage/St/2121 105414/JPN/2022	0.05962600
CrAssphage/WW/88U/SA/2022	Phage/St/3075/JPN/2023	0.06019881
CrAssphage/WW/112E/SA/2022	Phage/St/3575 41778/JPN/2023	0.06669287
CrAssphage/WW/111E/SA/2022	Phage/St/3075/JPN/2023	0.06953129
CrAssphage/WW/88U/SA/2022	Phage/St/3986 25839/JPN/2023	0.08131183
CrAssphage/WW/88U/SA/2022	Phage/St/3950 21566/JPN/2023	0.08131183
CrAssphage/WW/88U/SA/2022	Phage/St/2929 39558/JPN/2023	0.08131183
CrAssphage/WW/88U/SA/2022	Phage/St/2861 38114/JPN/2023	0.08131183
CrAssphage/WW/88U/SA/2022	Phage/St/2087 16664/JPN/2023	0.08131183
CrAssphage/WW/88U/SA/2022	Phage/St/PF-P005 31277/JPN/2023	0.08131183
CrAssphage/WW/88U/SA/2022	Phage/St/3316 61037/JPN/2022	0.08131183
